# Viral Activation of MK2-hsp27-p115RhoGEF-RhoA Signaling Axis Causes Cytoskeletal Rearrangements, P-body Disruption and ARE-mRNA Stabilization

**DOI:** 10.1371/journal.ppat.1004597

**Published:** 2015-01-08

**Authors:** Jennifer A. Corcoran, Benjamin P. Johnston, Craig McCormick

**Affiliations:** 1 Department of Microbiology and Immunology, Dalhousie University, Halifax, Nova Scotia, Canada; 2 Beatrice Hunter Cancer Research Institute, Halifax, Nova Scotia, Canada; University of Pennsylvania Medical School, United States of America

## Abstract

Kaposi's sarcoma-associated herpesvirus (KSHV) is the infectious cause of several AIDS-related cancers, including the endothelial cell (EC) neoplasm Kaposi's sarcoma (KS). KSHV-infected ECs secrete abundant host-derived pro-inflammatory molecules and angiogenic factors that contribute to tumorigenesis. The precise contributions of viral gene products to this secretory phenotype remain to be elucidated, but there is emerging evidence for post-transcriptional regulation. The Kaposin B (KapB) protein is thought to contribute to the secretory phenotype in infected cells by binding and activating the stress-responsive kinase MK2, thereby selectively blocking decay of AU-rich mRNAs (ARE-mRNAs) encoding pro-inflammatory cytokines and angiogenic factors. Processing bodies (PBs) are cytoplasmic ribonucleoprotein foci in which ARE-mRNAs normally undergo rapid 5′ to 3′ decay. Here, we demonstrate that PB dispersion is a feature of latent KSHV infection, which is dependent on kaposin protein expression. KapB is sufficient to disperse PBs, and KapB-mediated ARE-mRNA stabilization could be partially reversed by treatments that restore PBs. Using a combination of genetic and chemical approaches we provide evidence that KapB-mediated PB dispersion is dependent on activation of a non-canonical Rho-GTPase signaling axis involving MK2, hsp27, p115RhoGEF and RhoA. PB dispersion in latently infected cells is likewise dependent on p115RhoGEF. In addition to PB dispersion, KapB-mediated RhoA activation in primary ECs caused actin stress fiber formation, increased cell motility and angiogenesis; these effects were dependent on the activity of the RhoA substrate kinases ROCK1/2. By contrast, KapB-mediated PB dispersion occurred in a ROCK1/2-independent manner. Taken together, these observations position KapB as a key contributor to viral reprogramming of ECs, capable of eliciting many of the phenotypes characteristic of KS tumor cells, and strongly contributing to the post-transcriptional control of EC gene expression and secretion.

## Author Summary

We have only scratched the surface in understanding how viruses control host gene expression. Several viruses disrupt important sites of post-transcriptional control of gene expression known as processing bodies (PBs), but underlying regulatory mechanisms and biological relevance remain poorly understood in most cases. Our study shows that the Kaposin B (KapB) protein of Kaposi's sarcoma (KS)-associated herpesvirus, known to block the degradation of a class of labile host mRNAs, does so by constitutively activating a signaling axis involving MK2, hsp27, p115RhoGEF and RhoA, thereby dispersing PBs. Thus, PB disruption may support the secretion of host pro-inflammatory cytokines and angiogenic factors that underlies KS tumor formation. Furthermore, by activating RhoA, KapB also causes cytoskeletal rearrangements, accelerated cell migration and angiogenesis in an endothelial cell model. Our findings position KapB as a key contributor to viral reprogramming of endothelial cells.

## Introduction

Kaposi's sarcoma-associated herpesvirus (KSHV), a.k.a. human herpesvirus-8 (HHV-8) is the infectious cause of Kaposi's sarcoma (KS), the most common malignancy of untreated AIDS patients, and two rare lymphoproliferative disorders, multicentric Castleman's disease (MCD) and primary effusion lymphoma (PEL) [1]–[3]. Like all herpesviruses, KSHV establishes persistent, life-long infection of its human host. The primary proliferative elements of KS lesions are latently infected endothelial cells (ECs) with an abnormal spindle-shaped morphology, commonly known as ‘spindle cells’. In latency, the viral episome persists in a reversible latent state and viral gene expression is limited to 6 consensus protein products (LANA, v-cyclin, v-FLIP, Kaposins A, B, and C) and 12 pre-miRNAs that are processed into at least 17 mature miRNAs (reviewed in [4], [5], [6]). Spindle cells display actin cytoskeleton rearrangements, enhanced cell motility and an aberrant angiogenic phenotype (recently reviewed in [7], [8]); all of these features can be recapitulated during *in vitro* infection of primary ECs [8]–[11]. Several KSHV latent gene products have been shown to contribute to these dramatic alterations in EC physiology (reviewed in [8]), but our understanding of their relative contribution to tumor-initiating events remains incomplete.

During KSHV infection, a complex translational program involving translation initiation at non-canonical CUG codons and decoding sets of GC-rich repeats results in the generation of multiple kaposin protein products, including Kaposin B (KapB). We have previously shown that KapB regulates the expression of pathogenetically important pro-inflammatory cytokines and angiogenic factors at the post-transcriptional level [12]. This is achieved by direct binding and activation of mitogen-activated protein kinase (MAPK)-associated protein kinase-2 (MK2), a nodal kinase that regulates the turnover of mRNAs bearing AU-rich instability elements (AREs) within their 3′UTRs [13], [14]. AREs are commonly found in labile mRNAs encoding tightly regulated, potent effector molecules, including many cytokines, angiogenic factors and proto-oncogenes [15], [16]. MK2 phosphorylates a variety of target proteins, including several ARE-binding proteins (ARE-BPs) that govern ARE-mRNA stability, with the net effect of causing ARE-mRNA stabilization. For example, the ARE-BP tristetraprolin (TTP) normally promotes ARE-mRNA turnover by facilitating interactions between bound mRNAs and the cytoplasmic mRNA degrading enzymes associated within exosomes and processing bodies (p-bodies, PBs) (reviewed in [17]). Phosphorylation of TTP by MK2 creates a binding site for 14-3-3 scaffolding proteins, thereby preventing association of ARE-mRNAs with the decay machinery. Accordingly, MK2 activation during latent KSHV infection or in response to ectopic expression of KapB coincided with dramatic stabilization of ARE-mRNAs and increased production of a number of canonical products of ARE-mRNAs, including IL6 and CSF2 [12]. Importantly, KapB also stabilized the ARE-mRNA encoding PROX1 [18], a master regulator of lymphatic reprogramming of vascular endothelial cells, thereby providing a molecular mechanism for KSHV-mediated cell fate reprogramming [1]. Altogether, these findings suggest that KapB makes key contributions to the reprogramming of ECs in KS lesions.

PBs are small ribonucleoprotein (RNP) granules that contain the requisite enzymes to mediate rapid mRNA deadenylation, decapping and exonucleolytic degradation in a 5′ to 3′ direction [19]–[23]. PBs also contain RNA induced silencing complexes (RISC), translational repressors (rck/p54) and many RNA-binding proteins such as the ARE-BP, TTP. PBs are constitutively present in most cells, but they are also dynamic structures; PB number and size increase in response to a variety of environmental stresses, when 5′ to 3′-exonucleolytic decay is blocked, or when translation is inhibited [24]. PB formation involves aggregation of RNA binding proteins, utilizing mRNA itself as an organizing scaffold; as such, PBs disperse after treatment of cells with RNase [25]. Not exclusively sites of ARE-RNA decay, PBs have been shown to have important roles in nonsense-mediated decay, RNA interference, and they can also harbor translationally-silenced mRNAs that have the potential to escape PBs and resume translation [26], [27]. PBs have intimate links to both the microtubule and the actin cytoskeleton (reviewed in [28]). Stationary PBs associate with actin bundles while mobile PBs connect to the microtubule network [29]. They are linked to microtubules via the microtubule-associated protein nesprin-1 and travel along microtubules using the retrograde motor protein dynein [30], [31]. More recently, it was shown that PB accretion and ARE-mRNA turnover was modulated by the cytoskeletal regulator, RhoA GTPase (RhoA) [32]. Relatively little is known about how RhoA and other upstream signaling proteins govern PB assembly and function.

The Rho family of small GTPases (Rho, Rac and Cdc42) are molecular switches that cycle between an inactive GDP-bound configuration and an active GTP-bound form with the aid of guanine nucleotide exchange factors (GEFs) (reviewed in [33], [34]). RhoA regulates actin cytoskeleton dynamics to facilitate normal cell attachment, the formation of actin stress fibers, cell migration and angiogenesis (reviewed in [35]–[37]). Inactive cytosolic RhoA translocates to membranes upon activation by G-protein coupled receptors, that link the G-protein Gα13 to RhoA activation via numerous GEFs including p115RhoGEF [34], [38]–[40]. There, in its active conformation, RhoA can bind numerous downstream effectors. The most extensively studied RhoA effector is the Rho-associated kinase ROCK, a serine/threonine kinase with two isoforms, ROCK1 and ROCK2, bearing 64% overall sequence identity, and many overlapping activities [41]. Upon binding to RhoA, ROCK1/2 promote actomyosin contractility and stress fiber formation by phosphorylating target proteins including focal adhesion kinase (FAK), LIM kinase 1 (LIMK1), myosin light chain (MLC) and myosin phosphatase target (MYPT-1) [42]–[45]. The effects of MK2 are not limited to regulation of ARE-mRNA turnover; numerous studies have pinpointed a role for MK2 in the actin cytoskeletal remodeling required to promote EC migration and angiogenesis [46]–[51] [52], [53]. Active MK2 phosphorylates the small heat shock protein (hsp) 27 and suppresses its actin filament capping activity, releasing the molecule from the barbed end of the actin fiber to permit actin fiber growth [53]–[55]. MK2 also phosphorylates the serine/threonine kinase, LIM kinase 1 (LIMK1) [56], which subsequently phosphorylates and inactivates the actin-severing protein cofilin [45]. Thus, the available data indicates that both MK2 and RhoA support EC cytoskeletal rearrangements, migration and angiogenesis. However, to date, little is known about how these two pathways are functionally integrated.

By studying KSHV latency, we have elucidated the functional integration of the MK2 and RhoA signaling pathways in ECs. We show that Kaposin B activates a signaling axis involving MK2, hsp27, p115RhoGEF and RhoA. The consequences of activation of this pathway in ECs include the formation of actin stress fibers, increased cell migration and angiogenesis, and dispersal of PBs. Because PBs are important sites of ARE-mRNA decay, KapB-mediated PB dispersal supports its role in potent stabilization of ARE-mRNAs. Taken together, these observations position KapB as a key contributor to viral reprogramming of ECs, capable of eliciting many of the phenotypes characteristic of KS tumor cells, and strongly contributing to the post-transcriptional control of EC gene expression and secretion.

## Results

### KapB activates RhoA GTPase to stimulate actin stress fiber formation, endothelial cell migration and tubule formation

**Figure 1:**
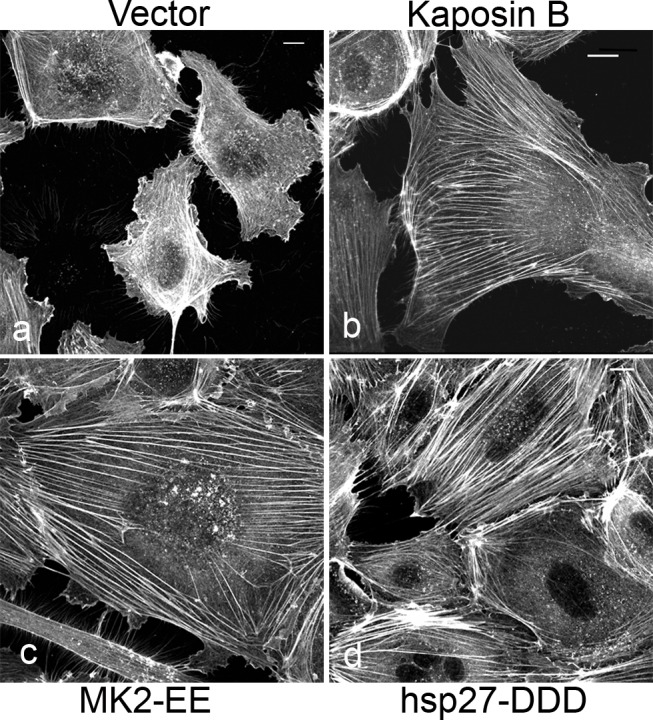
KapB promotes actin stress fiber formation in human umbilical vein endothelial cells. Expression of KapB (panel b) or controls (a constitutively active version of MK2 [MK2-EE, panel c], a phosphomimicking version of hsp27 [hsp27-DDD, panel d] or an empty vector control [panel a]) in HUVECs was achieved by retroviral transduction and a 2-day selection with puromycin, after which cells were split onto coverslips and processed for fluorescence microscopy. Cells were fixed in 4% paraformaldehyde (in PBS) permeabilized in 0.1% Triton X-100 (in PBS) and stained with Alexa 555-conjugated to phalloidin to visualize the actin cytoskeleton (panels a-d). MK2-EE and hsp27-DDD have previously been shown to induce actin stress fiber formation in endothelial cells [59]. Scale bar  = 10 µm.

Latent KSHV-infected ECs display marked alterations in cytoskeletal morphology. The latent vFLIP protein has been shown to modulate actin and cause spindling of ECs in an NF-kB-dependent manner [9], but the impact of the remaining latent gene products on the cytoskeleton remains largely unexplored. We previously reported that the latent KapB protein binds and activates MK2 kinase, known to be a major regulator of actin remodeling [12]. For this reason, we investigated the ability of KapB to modulate the actin cytoskeleton in human umbilical vein endothelial cells (HUVECs). Cells ectopically expressing KapB displayed thick parallel actin stress fibers (Fig. 1B). Actin stress fibers have been observed in cells following activation of the small GTPase RhoA [57], [58]. They have also been observed in cells with increased activation of the p38/MK2 signaling pathway or by expression of the constitutively active form of the kinase, MK2 (MK2-EE) or a phosphomimicking form of hsp27 (hsp27-DDD) [59] [60], [61]. Consistent with this, we observed that expression of either MK2-EE or hsp27DDD in HUVECs caused the formation of actin stress fibers (Figs. 1C, 1D). Activation of the p38/MK2 pathway by KapB also causes the increased secretion of pro-inflammatory cytokines that can then act in an autocrine or paracrine fashion to potentiate p38 pathway activation [12]. To address whether the appearance of actin stress fibers in response to KapB expression was mediated largely by KapB-mediated secretion of inflammatory molecules (ie. paracrine effects), we treated HUVECs with conditioned media from KapB-expressing cells. No stress fibers were observed in control cells treated with media from KapB-expressing cells (S1 Fig.), indicating that KapB-mediated rearrangement of the actin cytoskeleton was a cell autonomous effect.

**Figure 2:**
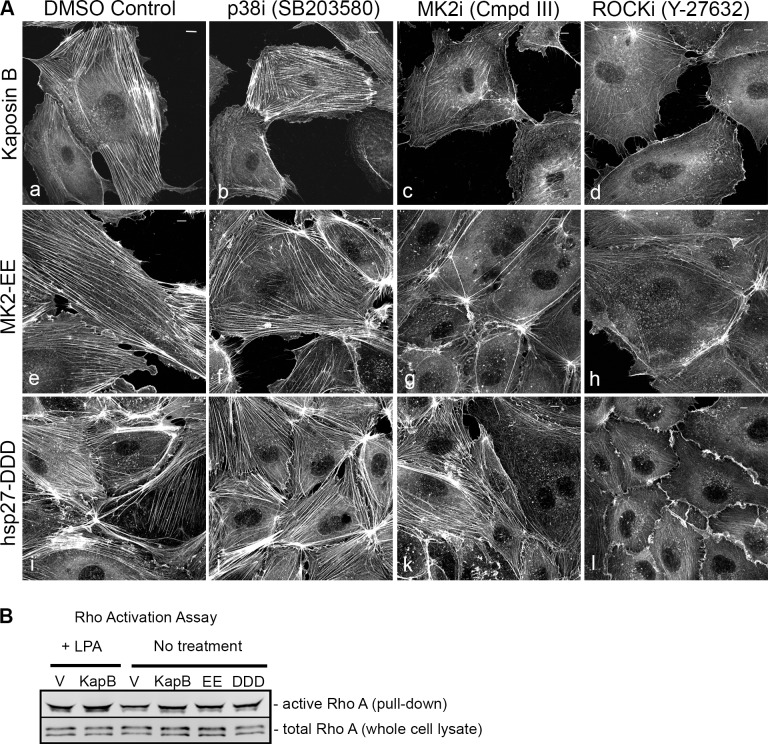
KapB requires the kinase MK2 and RhoA-GTPase to induce actin stress fibers in primary endothelial cells. A) HUVECs expressing KapB (panels a–d) or controls (MK2-EE [panels e–h] or hsp27-DDD [panels i–l]) were treated with inhibitors to examine the roles of the kinases p38 and MK2 and the GTPase RhoA in stress fiber formation. Cells were either treated for one hour with 3 µM of the p38 kinase inhibitor SB203580, 10 µM of the MK2 inhibitor, Inhibitor III, 10 µM of the rho-associated kinase (ROCK1/2) inhibitor, Y-27632, or DMSO as a vehicle control, before fixation and staining as above. B) To examine activation of the RhoA GTPase by KapB and controls, HeLa-Tet Off cells were transfected with an expression plasmids for KapB, MK2-EE, hsp27-DDD or an empty vector control and assayed for active (GTP-bound) RhoA using the Active Rho Detection Kit (CST) according to manufacturer's instructions. Before lysis, transfected cells were starved in low serum media for a total of 24–28 hours and either treated or not treated with the RhoA activator lysophosphatidic acid (LPA) for 3 minutes. Total cell lysate and pull-downs containing the active (GST-bound) form of RhoA were subjected to SDS-PAGE and immunoblotted with anti-RhoA (CST). One representative experiment of three is shown.

**Figure 3:**
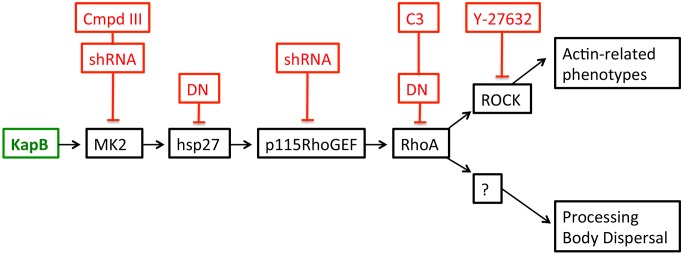
KapB activation of MK2 can lead to RhoA through intermediates hsp27 and p115RhoGEF. A recently described signaling pathway links activated MK2 to RhoA through intermediates hsp27 and p115RhoGEF (63). Hsp27 phosphorylation by MK2 is an important ‘switch’ that allows local recruitment of p115RhoGEF to a signaling complex, promoting RhoA activity. We hypothesize that KapB mediated activation of this pathway contributes to several downstream effects of active RhoA.To address the importance of individual proteins within this signaling pathway in the mechanism of KapB-mediated RhoA activation, PB dispersion and actin cytoskeletal rearrangements, we undertook several approaches to eliminate expression or activity of candidate proteins. These included the co-expression of dominant negative (DN) versions of RhoA and hsp27, co-expression of shRNAs designed to reduce expression of p115RhoGEF and MK2 using RNA interference, and chemical inhibitors that were specific for RhoA (C3) and its downstream effector kinase ROCK (Y-27632).

Selective chemical inhibitors were used to investigate the mechanism of KapB-mediated actin rearrangements. Treatment of KapB-expressing HUVECs with a selective MK2 inhibitor [62] prevented formation of actin stress fibers, whereas p38 MAPK inhibition had no effect (Fig. 2A). This data is consistent with the notion that KapB binds and activates MK2 downstream of p38 MAPK. Treatment of cells with a selective inhibitor of the Rho kinases ROCK1 and ROCK2 (hereafter referred to as ROCK), which are RhoA substrates known to play a role in actin stress fiber formation, also inhibited KapB-mediated stress fibers (Fig. 2A). Taken together, these data suggest that KapB-mediated actin rearrangements depend upon activity of both MK2/hsp27 and RhoA/ROCK signaling axes. Because previous studies suggested a functional link between MK2/hsp27 and RhoA activation [63], we measured RhoA activity in KapB-expressing cells using a pull-down assay that isolates only the active (GTP-bound) form of RhoA [64]. KapB expression activated RhoA, both in the absence (Fig. 2B, lane 4) and presence (Fig. 2B, lane 2) of the canonical RhoA activator, LPA [65]. Interestingly, we also observed increased pull-down of the active form of Rho from cells transfected with MK2-EE and hsp27-DDD (Fig. 2B, lanes 5 and 6). Thus, RhoA was activated in cells where MK2 activity was mimicked or stimulated by direct KapB-MK2 binding. This is consistent with previous reports of a non-canonical MK2/hsp27/p115RhoGEF/RhoA signaling pathway in arachadonic acid-treated epithelial cells (63) (Fig. 3).

In addition to spurring actin stress fiber formation, activation of Rho family GTPases and the p38/MK2/hsp27 MAPK pathway has previously been linked to increased cell migration, and in the case of ECs, increased angiogenesis [33], [49]–[51], [56], [60]. We observed that ectopic KapB expression in HUVECs promoted cell migration in a wound-healing assay; KapB-expressing cells displayed 59% wound closure compared to 25% wound closure by control cells over a 6-hour period (S2A Fig.). KapB also promoted migration of HUVECs across a gelatin-coated semi-permeable membrane (S2B Fig.). Interestingly, KapB-mediated enhancement of cell migration was detectable only in the absence of the potent endothelial angiogenic molecule, vascular endothelial growth factor (VEGF), though VEGF treatment has no appreciable effect on KapB expression level (S3 Fig.). The p38/MK2/hsp27 pathway plays a clearly defined role mediating the migration of ECs in response to VEGF [60]; however, our results suggest that the activation of this pathway by KapB also mediates EC migration when VEGF levels are low. This supports the notion that KapB targets nodal kinases commonly stimulated during EC migration.

Endothelial cells form tubules on matrigel in an *in vitro* angiogenesis assay that mimics the formation of blood vessels [56], [66]. We examined the effect of KapB expression on tubule formation compared to control HUVECs expressing either empty vector or the constitutively active from of MK2 (MK2-EE). Both KapB- and MK2-EE-expressing HUVECs formed tubules in matrigel (S4 Fig.). This is consistent with the well-described role of the p38/MK2 pathway in promoting EC tubule formation [50], [56]. In both cases, tubule network formation was reduced after treatment with the ROCK inhibitor Y-27632 (S4B Fig.). Thus, by activating MK2 and RhoA, KapB deregulates several processes in primary ECs contributing to a migratory and angiogenic phenotype.

### KapB causes p-body dispersion in a RhoA-dependent manner

**Figure 4:**
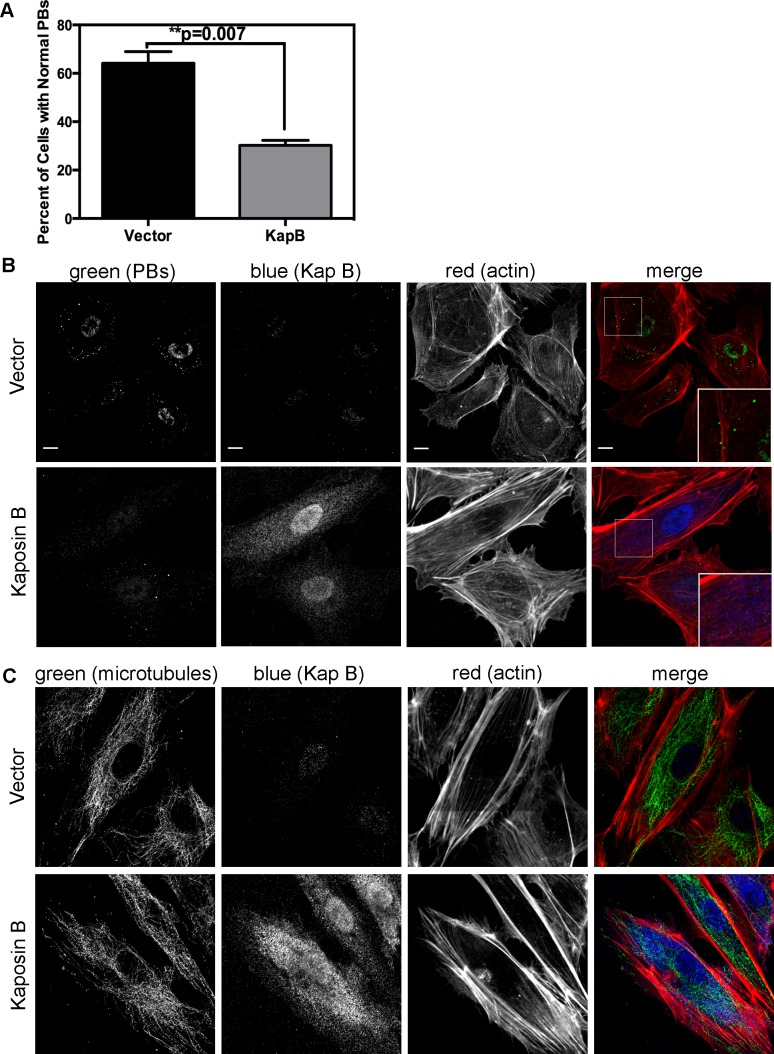
KapB disperses p-bodies. A–B) HUVECs transduced to express of KapB or vector control were split onto coverslips and processed for immune fluorescence. Cells were fixed 4% paraformaldehyde (in PBS) permeabilized in 0.1% Triton X-100 (in PBS), and blocked in 1% human AB serum before staining with the following primary antibodies: anti-hedls (p-bodies, green) and anti-KapB (KapB, false-coloured blue). To visualize actin stress fibers, cells were also labeled with Alexa 647-conjugated phalloidin (red). To quantify the effect on KapB expression on p-bodies, the number of KapB-expressing cells that displayed p-bodies of normal size (>300 nm in diameter) was counted and compared to control cells. Five independent experiments were performed. >100 cells were counted in each experiment. Scale bars  = 10 µm. C) To stain the microtubule cytoskeleton, cell fixation was performed in pre-warmed, 37°C 4% paraformaldehyde (in D-PBS) for 10 minutes, before permeabilization and immunostaining using anti-KapB (false-colored blue) or anti-tubulin (microtubules, green). Scale bars  = 10 µm.

KapB stabilizes labile host cell ARE-mRNAs. Interestingly, a known site of ARE-mRNA translational repression and degradation is the processing body (PB), which has intimate links to both the actin and the microtubule cytoskeleton (reviewed in [28]); stationary PBs associate with actin bundles while mobile PBs connect to the microtubule network [29]. More recently, it was shown that RhoA GTPase activity modulates PB formation [32]. Because KapB activates RhoA we reasoned that PB disruption might contribute to KapB-mediated ARE-mRNA stabilization. PBs were visualized by immunofluorescent staining for two PB resident proteins, hedls or DDX6 [67]. Indeed, KapB-expressing HUVECs displayed a decrease in the number of cells containing PBs of expected dimensions (approximately 0.3 µm in diameter, [68]) (Fig. 4A–B). This effect was quantified by counting the number of KapB-expressing cells that contained one or more PBs of normal size compared to control (empty vector) transduced cells. We observed that in control cell populations, 64% of cells contained normal PBs, compared to 30% in KapB-expressing cells. HUVECs were also stained for the actin cytoskeleton, and KapB-expressing cells displayed thick parallel actin stress fibers (Fig. 4B), consistent with our previous observations that KapB induces actin polymerization and RhoA activation (Figs. 1–2).

It is not known how RhoA activation modulates PBs. RhoA GTPase can be activated by the microtubule-bound guanine exchange factor GEF-H1; microtubule disruption causes release of GEF-H1 and concomitant RhoA activation [69]. To test whether KapB-mediated activation of RhoA and disruption of PB accretion were related to a disruption of the microtubular network, we stained KapB-expressing HUVECs for α-tubulin. We did not observe altered tubulin staining intensity nor did we observe any striking differences in the appearance of the microtubule network in cells expressing KapB compared to controls (Fig. 4C), indicating that KapB-mediated suppression of PBs is independent of changes to microtubule cytoskeleton, and suggesting that RhoA activation is unlikely a result of the release of GEF-H1 from microtubules.

**Figure 5:**
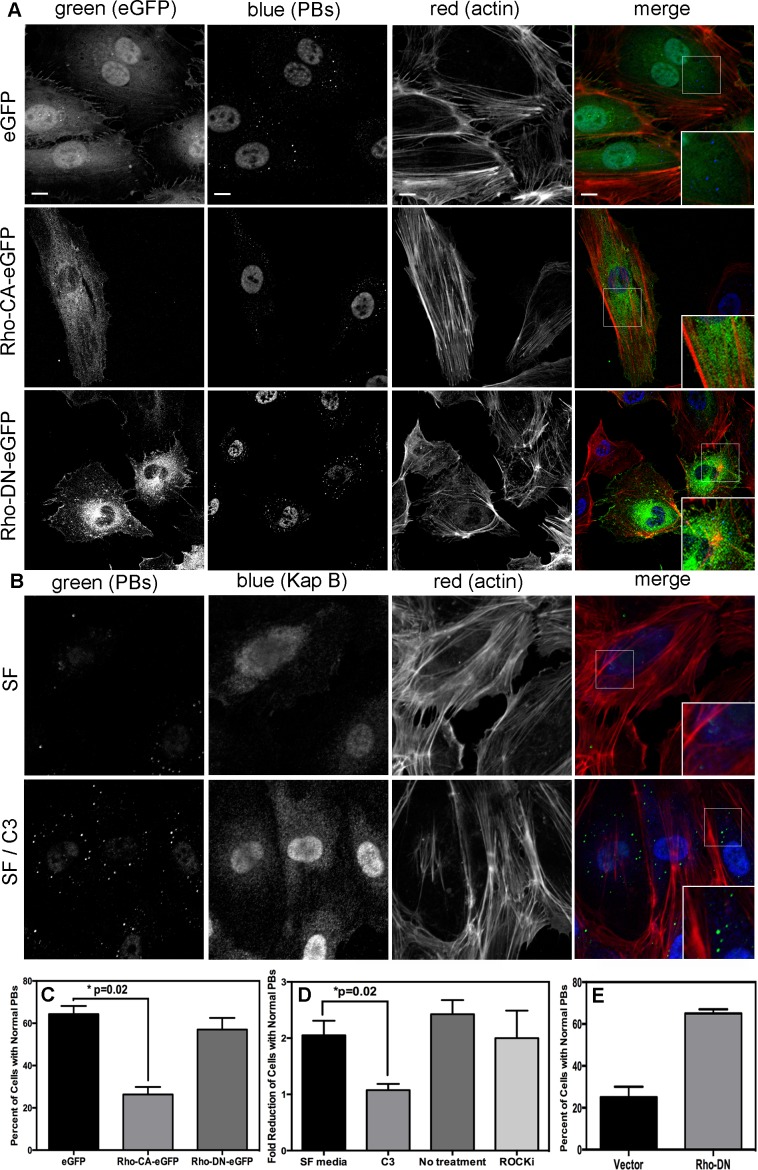
KapB activation of RhoA-GTPase but not the downstream kinase ROCK is necessary for p-body disruption. A-D) HUVECs were transduced with recombinant retroviruses to express eGFP, a constitutively active version of Rho (RhoCA-eGFP), a dominant negative version of Rho (RhoDN-eGFP), KapB, or empty vector. Following two-day selection with puromycin, cells were seeded onto coverslips. The next day, cells were either not treated or treated with C3 transferase (in basal media) for 6 hours at 37°C to irreversibly inactivate Rho GTPase. After the 6-hour incubation, cells received one hour of normal media before fixation, permeabilization, and staining as described for Fig. 4. Alternately, cells were treated with the specific inhibitor of the Rho kinase ROCK 1/2 (10 µM of Y-27632) for one hour at 37°C before immunostaining. To quantify p-body disruption, the number of cells expressing KapB or controls that retained normal p-bodies was counted as for Fig. 4. n = 3 independent experiments; Scale bar  = 10 µm. E) HUVECs were sequentially transduced with two populations of recombinant retroviruses: firstly, puromycin-resistant viruses that express KapB or the empty vector; and secondly, blasticidin-resistant viruses that express a dominant negative version of Rho (Rho DN-HA tagged) or the empty vector. At each step, positive transductants were selected with the appropriate drug for 2 days. Following this, cells were seeded onto coverslips and the next day, treated with basal media for 1 hour before fixation, permeabilization and staining as described. To quantify p-body disruption, the number of cells expressing the transgene of interest that retained normal p-bodies was counted as for Fig. 4 (n = 2 independent experiments).

When Takahashi *et al.* [32] observed that the overexpression of RhoA mediated an alteration to PB dynamics, they concluded that RhoA induced an increase in the number of PBs while causing a marked reduction in average PB size. Using retroviral transduction, we expressed a constitutively active (CA) version or dominant negative (DN) version of RhoA fused to GFP (Rho-CA-eGFP or Rho-DN-eGFP) in primary ECs [37], [70]. We observed both a marked loss of total PBs and a reduction in remaining PB size in HUVECs expressing Rho-CA-eGFP, whereas PBs in cells expressing Rho-DN-eGFP were similar to eGFP control (Fig. 5A). This is consistent with our previous observations that chemical activators of RhoA, including LPA and nocodazole, disrupted PB accretion [67]. Cells expressing CA-RhoA-eGFP also display marked actin stress fiber formation that is lacking in control cells or cells expressing the Rho-dominant negative (DN) construct [37], [70] (Fig. 5A). Irreversible inactivation of RhoA by C3 transferase, which ADP-ribosylates the Asn41 residue, disrupts RhoA-mediated re-organization of actin filaments [38], [71]. To determine whether RhoA activity is required for KapB-mediated actin polymerization and disruption of PBs, we treated KapB-expressing HUVECs with C3. In the absence of C3, the number of cells containing normal PBs was reduced two-fold by KapB expression; when treated with C3, this number was restored to control levels (Fig. 5B, D). C3 also abrogated the ability of KapB to promote actin stress fibers. We also co-expressed KapB with RhoA-DN by sequential transduction of HUVECs. Like C3 treatment, Rho-DN expression also prevented the ability of KapB to disperse PBs (Fig. 5E). However, when KapB-expressing HUVECs were treated with ROCK inhibitor there was no effect on KapB-mediated PB disruption (Fig. 5D). By contrast, ROCK inhibition prevented KapB-mediated stress fiber formation (Fig. 2). This observation uncouples the effect that RhoA activation has on PBs from canonical RhoA effects on actin and migration - processes that clearly require ROCK. Taken together, these experiments show that KapB-mediated PB disruption is dependent on RhoA, but not ROCK, consistent with previous findings [32] (Fig. 3).

### KapB activation of MK2 is required for RhoA activity and PB disruption

**Figure 6:**
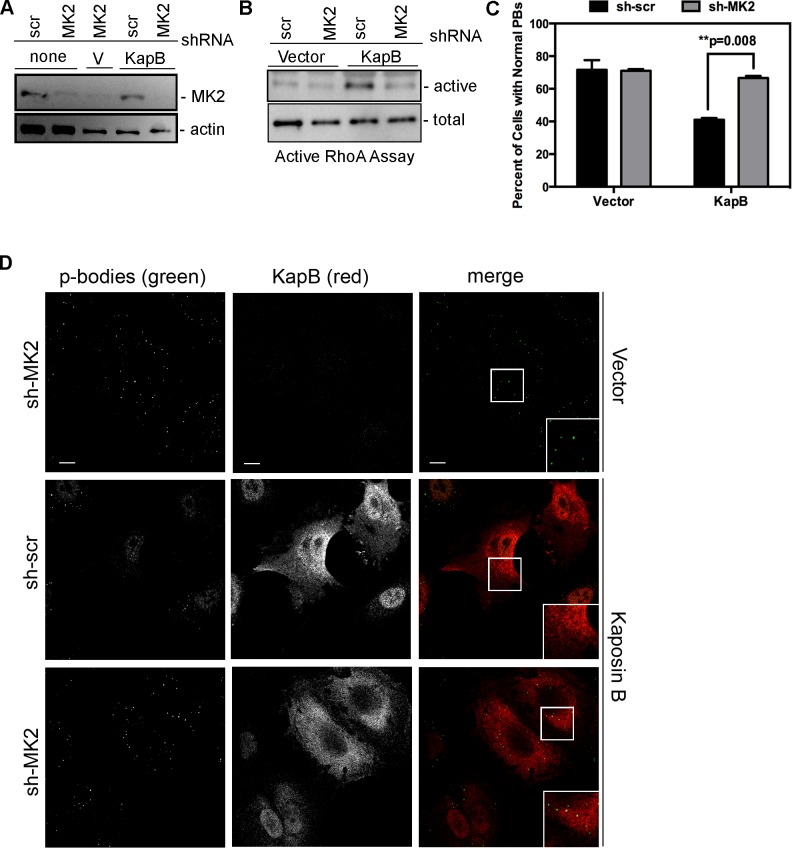
KapB-mediated activation of RhoA-GTPase and PB dispersal requires MK2. HUVECs were sequentially transduced with two populations of recombinant retroviruses: firstly, puromycin-resistant viruses that express either a short hairpin RNAs (shRNAs) against MK2 or the scrambled (scr) shRNA control, and secondly, viruses that express KapB or the empty vector. After the first step, positive transductants were selected with puromycin for 2 days. A) To examine MK2 expression after knock-down, HUVECs were washed with PBS and lysed in 1x SDS-protein sample buffer containing protease inhibitors and processed for SDS-PAGE and immunoblotting using anti-MK2 and anti-beta-actin. One representative blot of two is shown. B) To assess RhoA activity, transduced HUVECs were assayed for active (GTP-bound) RhoA using the Active Rho Detection Kit (CST) according to manufacturer's instructions. Confluent 6-well plates of HUVECs cells were used for the assay. Total cell lysate (1/10) and pull-downs containing the active (GST-bound) form of RhoA were subjected to SDS-PAGE and immunoblotted with anti-RhoA. One representative experiment is shown. C–D) To examine PBs, transduced cells were seeded onto coverslips. KapB-expressing cells were identified by positive immunoflurescent staining with anti-KapB. To quantify p-body disruption, the number of cells expressing KapB that retained normal p-bodies was counted as for Fig. 4 (n = 3 independent experiments; Scale bar  = 10 µm).

We hypothesized that KapB expression activates a little-known signaling axis that links upstream activation of the p38/MK2 MAPK pathway to RhoA activity (Fig. 3) (63). To examine the role of MK2 in KapB-mediated RhoA activation, we generated retroviruses expressing short hairpin (sh)-RNAs directed against MK2. Efficient silencing of endogenous MK2 expression (Fig. 6A) prevented RhoA activation in KapB-expressing HUVECs (Fig. 6B). Furthermore, MK2 silencing prevented efficient KapB-mediated PB dispersion (Figs. 6C–D). These data confirm a role for MK2 in the mechanism of KapB-mediated RhoA activation and PB dispersion.

### KapB-mediated RhoA activation contributes to AU-rich mRNA stabilization

**Figure 7:**
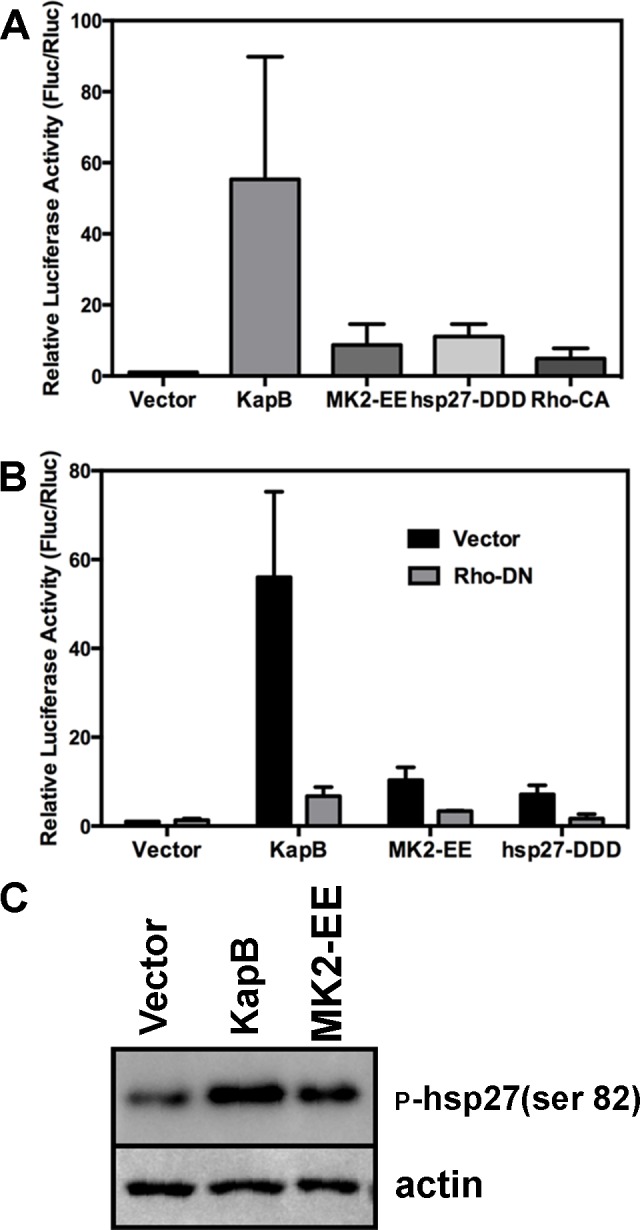
KapB-mediated activation of RhoA-GTPase is important for stabilization of ARE-containing mRNA. A) HeLa Tet-Off cells were co-transfected with pTRE2-Rluc (no ARE), pTRE2-Fluc-ARE, and an expression plasmids for KapB, the constitutively active forms of RhoA (Rho CA) and MK2 (MK2-EE), the phosphomimicking form of hsp27 (hsp27-DDD) or an empty vector control. At 24 hours post transfection, Dox was added to halt reporter gene transcription. 24 hours after Dox addition, cell lysates were harvested and normalized (firefly/renilla activity) luciferase activity was calculated. Results are displayed in relative light units (RLUs) and are the average of three independent experiments +/− the standard error. B) HeLa tet-off cells were co-transfected with pTRE2-Rluc (no ARE), pTRE2-Fluc-ARE, and an expression plasmids for KapB, the constitutively active form of MK2 (MK2-EE), the phosphomimicking form of hsp27 (hsp27-DDD) or an empty vector control. Transfections were performed with or without the addition of an expression plasmid for the dominant negative form of RhoA (Rho DN). At 24 hours post transfection, Dox was added to halt reporter gene transcription. 24 hours after Dox addition, cell lysates were harvested and analyzed for normalized (firefly/renilla activity) luciferase activity according to the methods. Results are displayed in relative light units (RLUs) and are the average of three independent experiments +/− the standard error. C) HUVECs were transduced with recombinant retroviruses to express KapB, MK2-EE, or empty vector, and selected with puromycin. 24 hours after selection, transduced cells were washed with PBS and lysed in 1x SDS-protein sample buffer containing protease inhibitors and processed for SDS-PAGE and immunoblotting using anti-phosphorylated (serine 82) hsp27 and anti-actin. One representative blot of three independent experiments is shown.

McCormick and Ganem [12] previously demonstrated that KapB expression caused a dramatic stabilization of AU-rich element (ARE)-mRNAs. To examine the role of RhoA in ARE-mRNA turnover, we utilized a reporter assay developed in our lab and described in detail in [72]. Briefly, HeLa Tet-Off cells were co-transfected with an empty plasmid vector or KapB expression vector, along with a doxycycline (dox)-responsive reporter plasmid encoding firefly luciferase linked to a canonical ARE derived from the labile CSF2 transcript. After 24 hours, transcription was arrested by the addition of dox, and 24 hours later, lysates were harvested for luciferase assays. Co-transfected dox-responsive Renilla luciferase reporter lacking an ARE served as a normalization control. KapB expression caused a striking increase in normalized luciferase activity, as did expression of MK2-EE, hsp27-DDD and RhoA-CA (Fig. 7A). These results are consistent with previous reports of control of ARE-mRNA turnover by RhoA [32] and p38/MK2 signaling pathways [13], [14], [73]. To further elucidate the role of RhoA in ARE-mRNA turnover, dominant negative RhoA (RhoA-DN) was introduced into this system. When KapB, MK2-EE and hsp27-DDD were co-expressed with RhoA-DN, the normalized luciferase activity was markedly reduced, indicating reduced stability of the firefly luciferase transcript (Fig. 7B). Phosphorylation of MK2 substrate hsp27 in cells expressing KapB or MK2-EE was confirmed by immunoblotting (Fig. 7C). These data suggest that KapB requires RhoA activation in order to achieve maximal ARE-RNA stabilization; furthermore, they confirm that MK2 activation precedes RhoA activation (Fig. 3). Thus, KapB causes ARE-mRNA stabilization via the direct binding and activation of MK2 which in turn causes RhoA activation.

### Constitutively active MK2 and hsp27 mediate PB dispersal by activating RhoA

**Figure 8:**
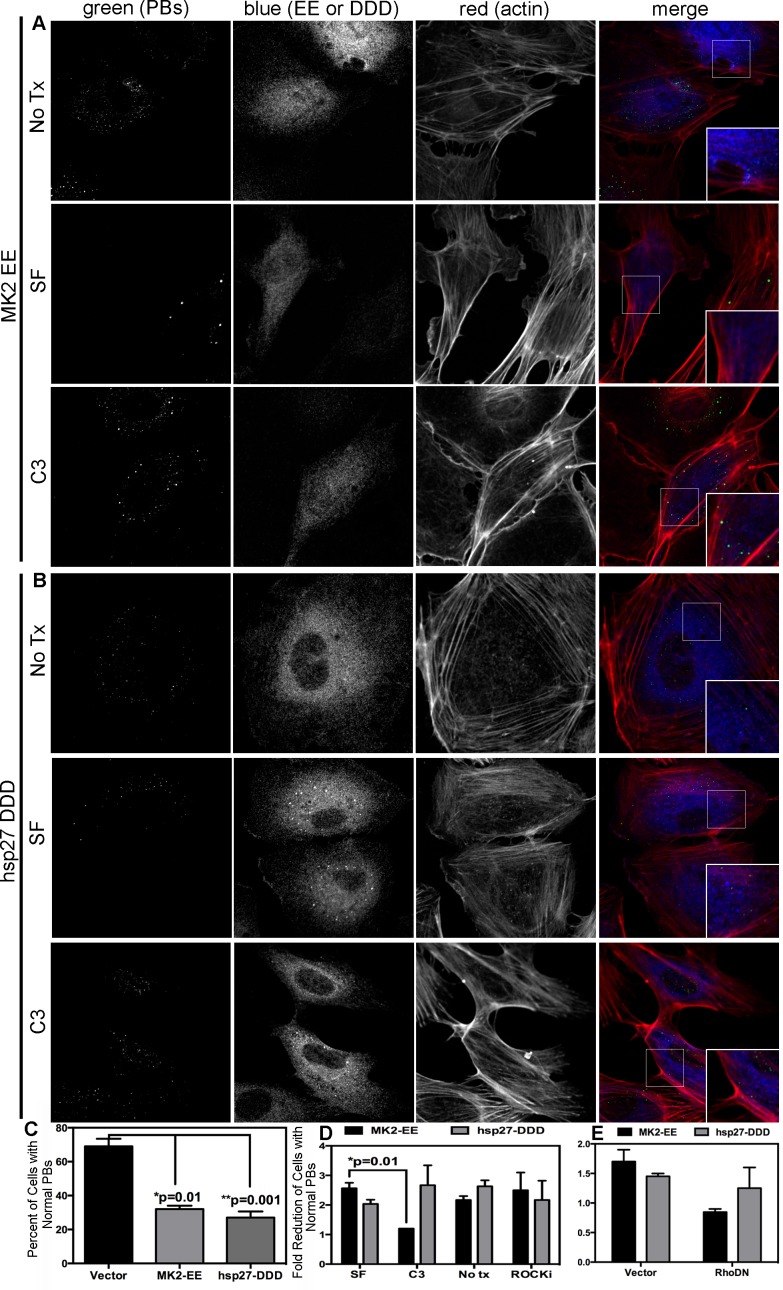
Upstream activators of Rho GTPase (MK2 EE and hsp27 DDD) disrupt p-bodies. A–D) HUVECs were transduced with recombinant retroviruses that express a constitutively active version of MK2 (MK2 EE, Flag-tagged) or phosphomimicking heat shock protein (hsp)27 (hsp27 DDD, HA-tagged). Following two-day selection with puromycin, cells were seeded onto coverslips. The next day, cells were either not treated or treated with C3 transferase (in basal media) for 6 hours at 37°C to irreversibly inactivate Rho GTPase. After the 6-hour incubation, cells received one hour of normal media before fixation, permeabilization, and staining with the following primary antibodies: mouse anti-hedls or rabbit anti-DDX6 (p-bodies, green), rabbit anti-HA (hsp27 DDD-HA, false-colored blue), or mouse anti-Flag (MK2 EE-Flag, false-colored blue). To visualize actin stress fibers, cells were also labeled with phalloidin (red). To quantify p-body disruption, the number of cells expressing MK2 EE or hsp27 DDD that retained normal p-bodies was counted as for Fig. 4. n = 3 independent experiments Scale bar  = 10 µm. E) HUVECs were sequentially transduced with two populations of recombinant retroviruses: firstly, puromycin-resistant viruses that express MK2 EE-Flag, hsp27 DDD-HA or the empty vector; and secondly, blasticidin-resistant viruses that express a dominant negative version of Rho (Rho DN-HA tagged) or the empty vector. At each step, positive transductants were selected with the appropriate drug for 2 days. Following this, cells were seeded onto coverslips and the next day, treated with basal media for 1 hour before fixation, permeabilization and staining as described. To quantify p-body disruption, the number of cells expressing the transgene of interest that retained normal p-bodies was counted as for Fig. 4 (n = 2 independent experiments).

Previous work demonstrated that p38/MK2-mediated RhoA activation depends on the phosphorylation of hsp27 on serines 15, 78 and 82, and the formation of a complex comprising phosphorylated hsp27 (p-hsp27), RhoA and the guanine exchange factor (GEF), p115RhoGEF [63]. Therefore, we hypothesized that KapB-mediated RhoA activation may require p-hsp27 and p115RhoGEF. To test this, we undertook a series of experiments that examined the importance of hsp27, p115RhoGEF and RhoA in modulating PB and actin dynamics in response to upstream activators. Expression of MK2-EE and hsp27-DDD in HUVECs promoted the disruption of PBs (Fig. 8A–C). Consistent with our hypothesis, we found that inhibition of RhoA using C3 transferase or co-expression of RhoA-DN restored PB levels to that of controls in MK2-EE-expressing HUVECs (Fig. 8A, D, E). However, when RhoA was inhibited (by either C3 or RhoA-DN) in hsp27-DDD-expressing HUVECs, PB levels were not restored (Fig. 8B–E). Treatment of MK2-EE- and hsp27-DDD-expressing HUVECs with the ROCK inhibitor had no effect on PB disruption (Fig. 8D), as previously observed for KapB (Fig. 5D).

**Figure 9:**
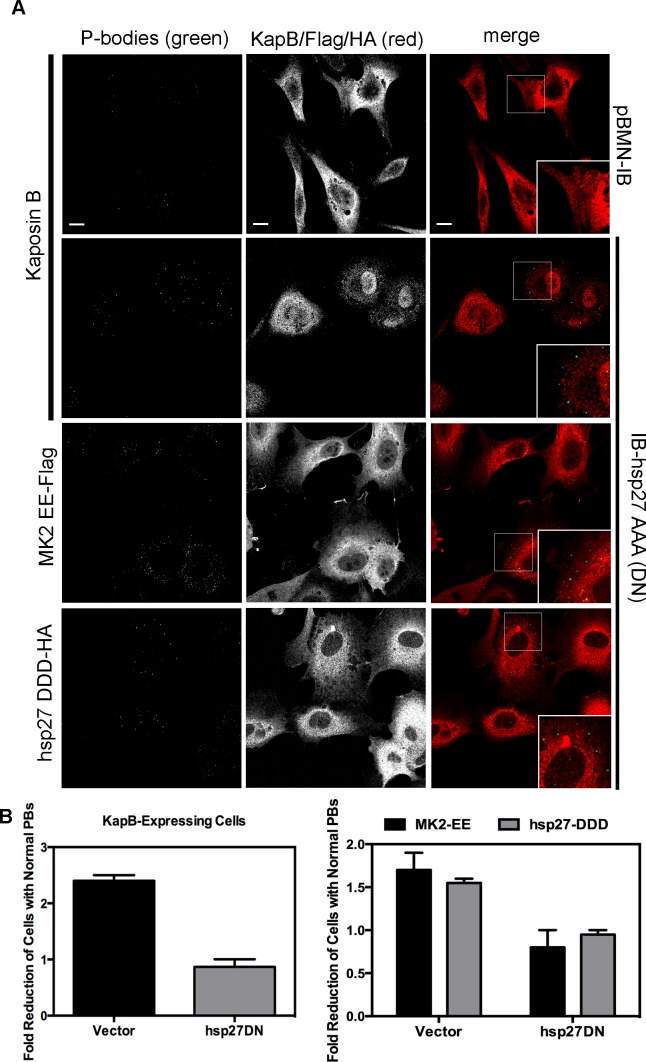
Dominant negative hsp27 inhibits KapB-induced p-body disruption. A–B) HUVECs were sequentially transduced with two populations of recombinant retroviruses: firstly, puromycin-resistant viruses that express KapB, MK2 EE-Flag, hsp27 DDD-HA or the empty vector; and secondly, blasticidin-resistant viruses that express a dominant negative version of hsp27 (hsp27 AAA-HA tagged) or the empty vector. At each step, positive transductants were selected with the appropriate drug for 2 days. Following this, cells were seeded onto coverslips and the next day, treated with basal media for 1 hour before fixation, permeabilization and staining with the following primary antibodies: mouse anti-hedls or rabbit anti-DDX6 (p-bodies, green), rabbit anti-KapB (red), rabbit anti-HA (hsp27-DDD-HA, red), or mouse anti-Flag (MK2-EE-Flag, red). Representative IF images are shown in A. To quantify p-body disruption, the number of cells expressing KapB, MK2-EE or hsp27-DDD that retained normal p-bodies was counted as described for Fig. 4 and shown in B (n = 2 independent experiments). Scale bar  = 10 µm.

We reasoned that KapB and MK2 activate RhoA by mediating complex formation between p115RhoGEF, RhoA and p-hsp27 (Fig. 3) (63). To investigate the role of p-hsp27, we utilized a dominant negative form of hsp27 (hsp27-AAA) in which the three canonical phosphorylation sites (serines 15, 78, 82) had been mutated to alanines [74]. Hsp27-AAA was co-expressed with KapB, MK2-EE and hsp27-DDD in HUVECs. In all cases, expression of the hsp27-DN prevented PB disruption, restoring PBs to normal levels (Fig. 9A–C). These data support an important role for hsp27 phosphorylation in KapB-mediated activation of RhoA and resulting PB dispersion.

### Knockdown of the Rho guanine exchange factor (GEF) p115 prevents KapB-mediated p-body dispersion

**Figure 10:**
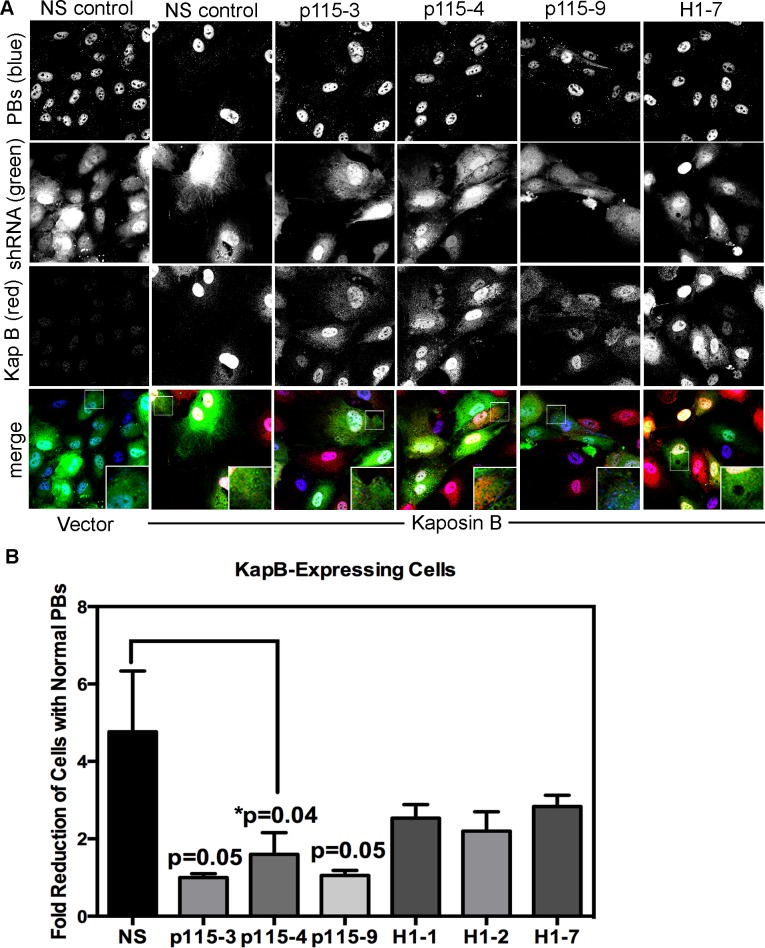
Knockdown of the Rho guanine exchange factor (GEF) p115 prevents KapB-induced modification of p-body dynamics. A-B) HUVECs were sequentially transduced with two populations of recombinant viruses: firstly, puromycin-resistant viruses that express KapB or the empty vector; and secondly, GFP-expressing lentiviruses that express short hairpin RNAs (shRNAs) against a two different Rho guanine exchange factors (GEFs; p115 [numbered −3, −4, and −9], H1 [numbered −1, −2, and −7], or the non-specific (NS) shRNA control. Positive transductants were selected by puromycin treatment (1^st^ step) and positive GFP-expression, to mark shRNA-expressing cells (2^nd^ step). After seeding cells on coverslips, and a one-hour treatment in basal media the following day, cells were stained with the following primary antibodies: mouse anti-hedls (to stain p-bodies, false-colored blue) and rabbit anti-KapB (to stain KapB, red). Representative IF images are shown in A. To quantify p-body disruption, the number of cells expressing the transgene of interest (red) and the shRNA construct (green) that retained normal p-bodies was counted as for Fig. 4 and shown in B (n = 3 independent experiments). Scale bar  = 10 µm.

Rho GTPase activity is regulated by numerous GEFs, which are in turn are regulated by upstream signals including G-protein activation (in the case of G-protein coupled receptors), phosphorylation (such as by receptor tyrosine kinases) or, as described by Garcia *et al.* [63], complex formation with phosphorylated hsp27. To confirm a role for the p115RhoGEF in our model of KapB-induced RhoA activation and PB disruption (Fig. 3), we generated lentiviruses expressing short hairpin (sh)-RNAs directed against p115RhoGEF and another RhoA GEF, namely GEF-H1. H1 is bound to microtubules and is released from upon their disruption (e.g. with nocodozole) to mediate activation of RhoA. When HUVECs were transduced with these lentiviral vectors, they expressed eGFP and displayed reduced expression of target genes (S5 Fig.). HUVECs expressing three different shRNA constructs targeting p115RhoGEF were unable to mediate PB disruption in cells expressing KapB, MK2-EE or hsp27-DDD (Fig. 10, S6 Fig.). By contrast, silencing of microtubule-bound GEF-H1 had no appreciable effect on PBs (Fig.10, S6 Fig.). This is consistent with our observation that microtubules are not disrupted in response to KapB expression (Fig. 4). Together, these data indicate that p115RhoGEF is essential for KapB-mediated RhoA activation and PB disruption.

### PBs are disrupted during latent KSHV infection

**Figure 11:**
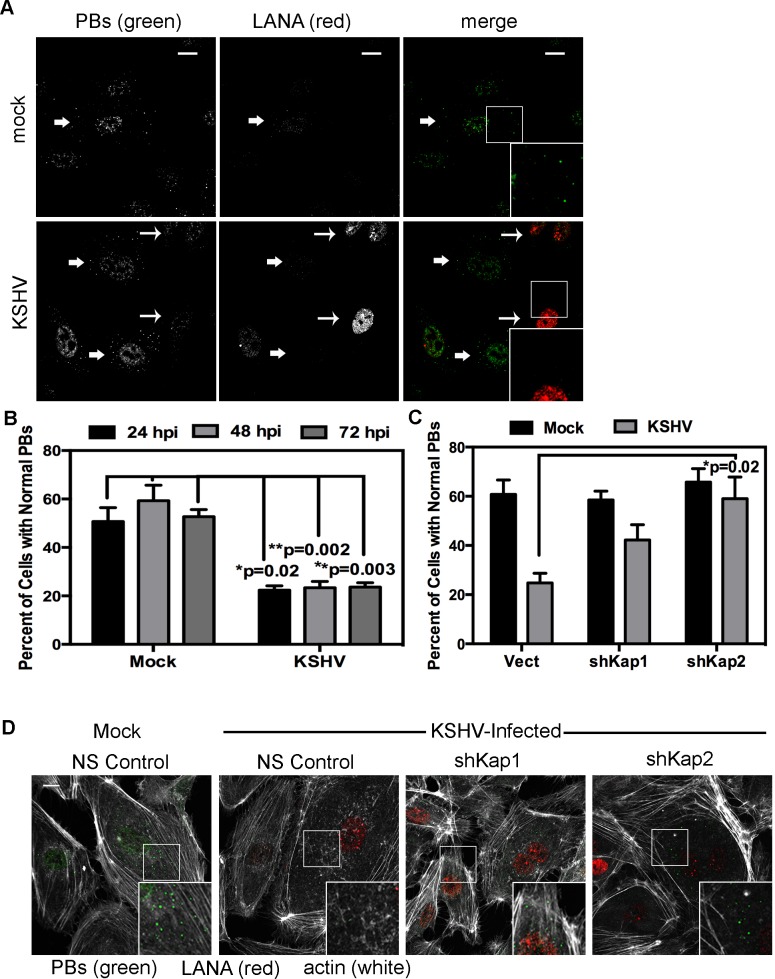
KSHV-mediated p-body dispersion in latently infected endothelial cells requires kaposin expression. A, D) HUVEC cells were either not infected or infected with KSHV for 24, 48, or 72 hours before being fixed with 4% paraformaldehyde. Cells were permeabilized in 0.1% Triton X-100 (in PBS), and blocked in 1% human AB serum before staining with the following primary antibodies: anti-hedls (p-bodies, green) and anti-LANA (to mark infected cells, red). In part A, infected cells are denoted with a thin arrow; uninfected cells are marked with a thick arrow. In part D, actin stress fibers were also labeled with phalloidin (false-colored white). B–C) To quantify the effect of latent infection on p-bodies, the number of LANA-expressing cells that displayed p-bodies of normal size (>300 nm in diameter) were counted and compared to uninfected cells. Three independent experiments were performed. >100 cells were counted in each experiment. C–D) Before KSHV infection HUVECs were transduced with two different recombinant lentiviruses that express a short hairpin RNAs (shRNAs) against the kaposin transcript (named shKap1 and shKap2) or the non-specific (NS) shRNA control. Positive transductants were selected by puromycin treatment for two days and then seeded on coverslips for infection with KSHV the next day. 48 hours post-infection, cells were fixed and processed for immune fluorescence as described above. n = 3 independent experiments Scale bar  = 10 µm.

**Figure 12:**
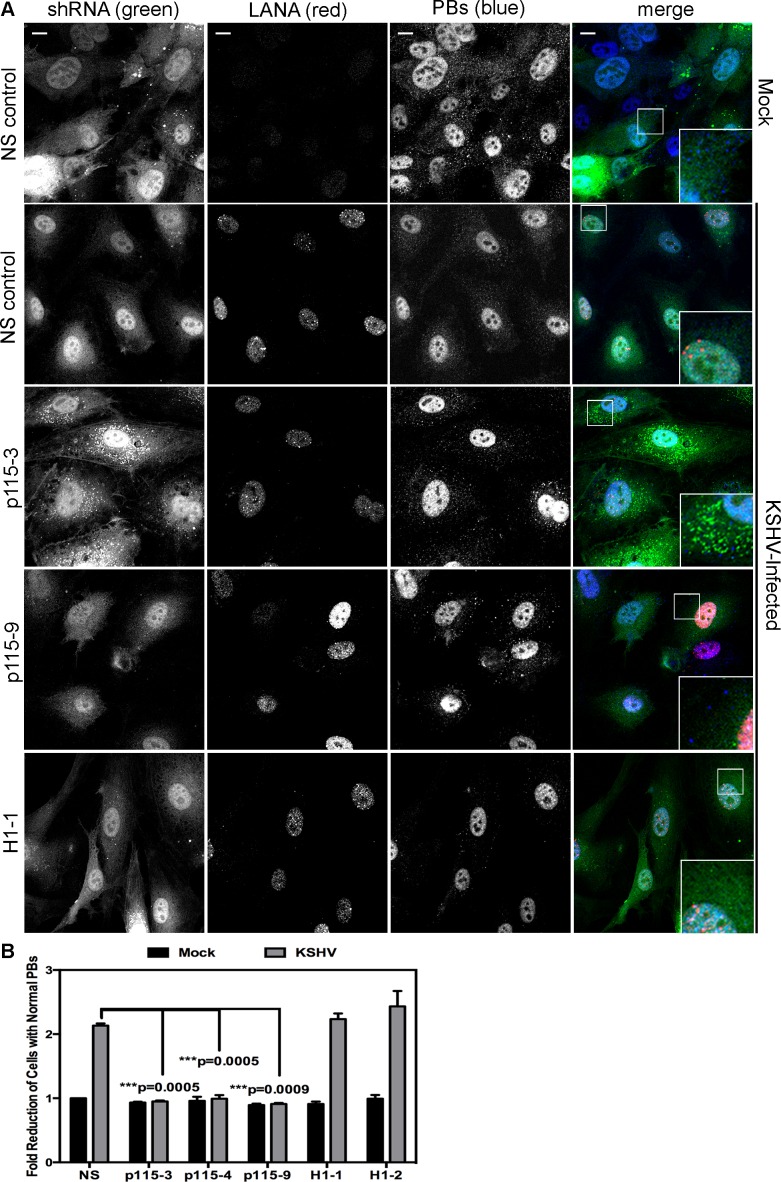
Knockdown of the Rho guanine exchange factor (GEF) p115 prevents KSHV-induced disruption of p-bodies in latently infected endothelial cells. A–B) HUVECs were transduced with recombinant GFP-expressing lentiviruses that express short hairpin RNAs (shRNAs) against the Rho guanine exchange factors (GEFs; p115 [numbered −3, −4, and −9] and H1 [numbered −1, −2]) or the non-specific (NS) shRNA control. Positive transductants were selected by puromycin treatment and positive GFP-expression. After seeding cells on coverslips for 24 hours, transduced HUVECs were either not infected or infected with KSHV for 48 hours before being fixed with 4% paraformaldehyde. Cells were permeabilized in 0.1% Triton X-100 (in PBS), and blocked in 1% human AB serum before staining with the following primary antibodies: anti-hedls (p-bodies, false-colored blue) and anti-LANA (to mark infected cells, red). Cells expressing the shRNA are GFP-positive (green). Representative IF images are shown in A. To quantify the effect of latent infection on p-bodies, the number of LANA-expressing cells that displayed p-bodies of normal size (>300 nm in diameter) was counted and compared to uninfected cells. Three independent experiments were performed and results are shown in B. Scale bar  = 10 µm.

HUVECs were infected with KSHV and establishment of latency was confirmed by LANA immunostaining (Fig. 11A). KapB expression during latent infection of HUVECs was confirmed by immunoblotting (S3 Fig.). LANA-positive cells displayed a marked reduction in the number of cells with normal-sized PBs at 24, 48 and 72 hours post-infection (Fig. 11 A, B). To implicate kaposin gene products in PB disruption, we transduced cells with shRNAs targeting the kaposin transcript (shKAP1, shKAP2). These shRNAs would be expected to silence expression of kaposin gene products (translated from spliced, cytoplasmic kaposin mRNA), but have no effect on *Drosha*-dependent processing of kaposin transcript-derived miRNAs in the nucleus. KSHV infection of cells bearing kaposin shRNAs revealed either a partial or full restoration in PB levels to that observed in uninfected cells (shKAP1 and shKAP2, respectively, Fig. 11C, D). These data indicate that at least one of the kaposin proteins is required for KSHV to alter PB dynamics during latent infection. Moreover, in parallel shRNA knockdown experiments we demonstrated that p115RhoGEF is essential for PB disruption in latently infected ECs, whereas GEF-H1 was dispensable (Fig. 12). Taken together, these findings indicate that during KSHV latency a product of the kaposin locus, likely KapB, activates the MK2-hsp27-p115RhoGEF-RhoA signaling pathway, thereby disrupting PBs.

## Discussion

**Figure 13:**
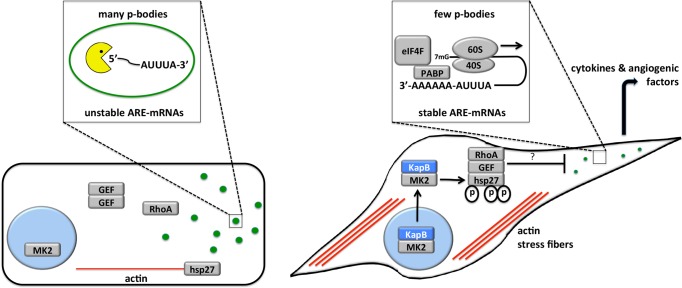
Model describing the effects of KapB in altering endothelial cell cytoskeleton and reprogramming gene expression by disrupting P-bodies and stabilizing ARE-mRNAs. When the p38/MK2 pathway is not activated by stress or KapB expression (cell on the left), MK2 remains in the nucleus, hsp27 remains bound to actin filaments, p115RhoGEF is found in a cytosolic oligomer-induced inhibitory state, and RhoA is not active. In this case, the actin cytoskeleton is unchanged, p-bodies are not dispersed and ARE-containing RNAs remain unstable. When KapB is expressed (cell on the right), it mimics the stress-induced activation of the p38/MK2 pathway, binds to MK2 and stimulates its kinase activity, resulting in hsp27 phosphorylation and the stimulation of p115RhoGEF activity, leading to RhoA activation. The consequences of the dual stimulation of RhoA and MK2 by KapB are as follows: 1. RhoA and ROCK-dependent formation of actin stress fibers, and migratory and angiogenic phenotype and 2. RhoA-dependent but ROCK-independent dispersal of PBs that correlates with the increased stability of ARE-containing mRNAs and the translation of their encoded proinflammatory and angiogenic protein products.

Latent KSHV infection of primary ECs *in vitro* causes dramatic changes in cellular physiology that largely reflect observations of KS tumor cells. Infected cells display marked alterations in signal transduction and gene expression, extended life span, and enhanced motility and angiogenic properties. Despite intensive efforts, the precise contributions of individual viral gene products to alterations in EC physiology remain incompletely understood. The data presented in the current study position KapB as a key contributor to viral reprogramming of ECs; ectopic expression of KapB leads to actin stress fiber formation and altered cell morphology, increased motility and an angiogenic phenotype; all of which are characteristic of the KS tumor cells (see model, Fig. 13). Moreover, KapB was sufficient to disrupt PBs, sites of mRNA translational repression and decay. These diverse phenotypes are linked to a signaling axis, comprising MK2, hsp27, p115RhoGEF and RhoA (Fig. 3), which likely evolved to respond to acute, transient stress and promote cell survival. By encoding the KapB protein that directly binds to the nodal kinase MK2, KSHV achieves constitutive activation of this pathway.

Several viruses have been shown to modulate PBs during infection, but the functional relevance of these observations are not yet clear (reviewed in [75]). Many viral RNAs associate with PB resident proteins [76] [77], and there have been reports of viral RNA recruitment to PBs [78]. Thus, PB dispersion might be an attractive mechanism for viral evasion of translational repression and RNA decay. Furthermore, some viruses have been shown to co-opt key PB resident proteins to support viral replication. For example, the flaviviruses West Nile virus and hepatitis C virus recruit DDX6 and Lsm1 to viral replication centers [79], [80] [81], and in so doing cause PB dispersion. By contrast, little is known about the impact of PBs on herpesvirus infection. Human cytomegalovirus has recently been shown to increase PBs in infected cells using a mechanism that requires active synthesis of cellular mRNA [82]. However, disruption of PB accretion by selective knock-down of several PB component proteins had no effect on virus replication in primary fibroblasts, making is unclear how enhanced PB formation benefits viral fitness.

We previously reported PB disruption during lytic KSHV infection, and demonstrated that the lytic viral gene vGPCR was sufficient to disrupt PBs in an ectopic expression model [67]. We now show that PB dispersal is also a cell autonomous feature of KSHV latency, and elucidate the molecular mechanism for KapB-mediated PB disruption. A common feature of these studies was strong correlation between PB dispersal and stabilization of endogenous [67] and exogenous ([67] and Fig. 7) ARE-containing RNAs, and resulting increases in protein production of the encoded products. Thus, multiple KSHV gene products converge on the regulation of ARE-mRNA turnover, providing an attractive mechanism for the overproduction of pro-inflammatory cytokines and angiogenic factors characteristic of KS lesions.

Our study reveals a previously unappreciated mechanism for the control of PB formation, involving MK2-mediated activation of RhoA. Our data fits well with a proposed model by Garcia *et al.* who observed that arachadonic acid-mediated activation of RhoA depended on prior activation of p38/MK2 and phosphorylation of the MK2 substrate hsp27 [63]. Hsp27 phosphorylation is an important ‘switch’ that allows local recruitment of p115RhoGEF and RhoA activation. In our experiments, we investigated the role of each of these signaling molecules in KapB-mediated PB dispersion. RhoA inhibition, either using the irreversible C3 transferase or expression of a dominant negative construct, blocked PB dispersion (Figs. 5, 8), as did the expression of a dominant negative version of hsp27 (Fig. 9). p115RhoGEF silencing using three different shRNA constructs also restored PB numbers to control levels in cells ectopically expressing KapB, MK2-EE, or hsp27-DDD or in latently KSHV-infected HUVECs (Figs. 10, 12, and S6 Fig.).

Ours is the first study to link MK2 to regulation of PBs, and confirm a previously identified role for RhoA [32]. However, the precise mechanism of PB dispersion downstream of RhoA activation remains to elucidated. Active RhoA signals through numerous effectors, the most extensively studied of which are the Rho-associated kinases (ROCKs), ROCK1 and ROCK2; binding of active RhoA relieves ROCK autoinhibition. PB disruption by KapB is insensitive to treatment with a ROCK inhibitor, suggesting that this process requires RhoA but not its downstream effector ROCK kinases (Figs. 5, 8). Overexpression of ROCK and another RhoA effector, the formin-family member mDia, likewise did not alter PBs [32]. PBs associate and traffic along microtubules [29], [83] and mDia activates microtubule polymerization and cause microtubule bundling [84], [85]. In isolation, these observations would make mDia an attractive candidate effector for PB dispersion. However, because the microtubule cytoskeleton is unchanged in KapB-expressing cells (Fig. 4), this suggests that KapB does not activate mDia. Moreover, shRNA knockdown of the microtubule-bound GEF-H1 had no effect on PB dispersion during latent KSHV infection or in cells ectopically expressing KapB (Figs. 10, 12), also suggesting that microtubules and mDia do not play a role in the mechanism of KapB-mediated PB dispersion. Future elucidation of the mechanism of MK2/hsp27/p115RhoGEF/RhoA-dependent PB dispersal will be challenging, but may be accelerated by careful inspection of PB resident proteins that might either be recruited by active RhoA, or phosphorylated by MK2.

Latent KSHV infection reprograms gene expression in ECs at transcriptional and post-transcriptional levels, but relative contributions of viral gene products to reprogramming remains incompletely understood. Our studies position Kaposin B as a chief post-transcriptional regulator of gene expression, binding and activating the nodal kinase MK2, thereby stabilizing and enhancing the translation of a variety of ARE-mRNAs encoding pathogenetically important pro-inflammatory cytokines and angiogenic factors. The effects of KapB on EC physiology are striking; formation of actin stress fibers, accelerated cell migration, and a strong angiogenic phenotype. Furthermore, KapB-mediated ARE-mRNA stabilization coincided with dispersal of PBs, a major site of ARE-mRNA decay in mammalian cells. By studying KapB, we gained new insight into the fundamental regulation of these processes by a recently identified signaling axis involving MK2, hsp27, p115RhoGEF and RhoA. Stress fiber formation, cell migration and angiogenesis were dependent on the activity of the RhoA substrate kinase ROCK, whereas PB dispersion occurred in a ROCK-independent manner. Taken together, these observations suggest that KapB is a key contributor to viral reprogramming of ECs, capable of eliciting many of the phenotypes characteristic of KS tumor cells, and strongly contributing to the post-transcriptional control of EC gene expression and secretion.

## Materials and Methods

### Reagents

Doxycycline (dox), blasticidin, puromycin, valproic acid, human AB serum, polyethyleneimine (PEI) and polybrene were purchased from Sigma-Aldrich Canada. C3 transferase (Rho inhibitor I) was from Cytoskeleton, Inc. MK2 inhibitor-III was purchased from Calbiochem. ROCK inhibitor Y-27632 was from Sigma.

To irreversibly inhibit RhoA-GTPase, HUVECs were treated with 1 µg/ml C3 transferase for 6 h in SF medium. Serum-free conditions are important to minimize baseline RhoA activity in the context of C3 exposure. However, since RhoA inhibition by C3 is irreversible after treatment, C3 and serum-free control cells were incubated for 1 h in normal medium to restore baseline PB levels.

### Cells

HeLa Tet-Off (Clontech), Phoenix-Amphotropic (a kind gift from G. Nolan, Stanford), and HEK293T cells (ATCC) were maintained at 37°C in a 5% CO_2_ atmosphere in Dulbecco's modified Eagle's medium containing 100 U of penicillin and streptomycin per ml and 10% heat-inactivated fetal bovine serum. Primary human umbilical vein endothelial cells (HUVECs) were purchased from Lonza. Cultures were expanded in EGM-2 medium (Lonza) on tissue culture plates coated with 0.1% (wt/vol) gelatin (in phosphate-buffered saline [PBS]) and used between passages 5 and 7 for experiments. The BCBL-1 primary effusion lymphoma (PEL) cell line was cultured in RPMI medium containing 10% heat-inactivated fetal bovine serum and 55 µM ß-mercaptoethanol.

### Plasmids

pcDNA3-MK2EE was a kind gift from Paul Anderson (Harvard University), and its creation is described in [86]. Briefly, to create pcDNA3-Flag-MK2-EE the cDNA encoding constitutively active murine MK2 was amplified with primers G42/G43 from pcDNA3mycMK2T205E/T317E [13]. The amplicons were digested with BamHI and XhoI, and inserted into the BamHI and XhoI sites of pcDNA3-Flag-BAK. An expression vector encoding the phosphomimicking version of small heat shock protein 27 (hsp27) in which serine residues at positions 15, 78 and 82 have been substituted with aspartic acid (pcDNA3.1-HAhsp27DDD) was generously obtained from Matthias Gaestel and its creation is described [54]. To generate the pcDNA3.1 HA-hsp27-AAA clone, two sequential reactions of Phusion site directed mutagenesis was performed on pcDNA3.1-HA-hsp27-DDD according to the instructions of the manufacturer (NEB) and using the following primers: Reaction 1. D15A forward 5′- GGCCCCGCCTGGGACCCC-3′, D15A reverse 5′- CCGCAGGAGCGAGAAGGGG-3′; Reaction 2. D78AD82A forward 5′- ACTCGCCAGCGGGGTCTCG-3′, D78AD82A reverse 5′- TGCCGGGCGAGCGCGCGG-3′. The expression vector for KapB (pCR3.1-kapB) and the ARE-RNA reporter plasmids (pTRE2-Rluc, pTRE2-Fluc-ARE, pTRE2-BBB, pTRE2-BBB-ARE, pTRE2-d1EGFP, and pTRE2-d1EGFP-ARE) have been previously described [12], [67], [72]. pCB6-eGFP-RhoA-CA and pCB6-eGFP-RhoA-DN plasmids were a generous gift from Dr. Roy Duncan, Dalhousie University.

### Retroviral expression plasmids

To create the pBMN-GFP-IP plasmid, the pEGFP-N1 plasmid (Clontech) was digested with NotI, subjected to a standard fill-in reaction with Klenow DNA polymerase (NEB), and further digested with BglII, releasing the eGFP ORF. To prepare the recipient pBMN-IP vector (G. Nolan lab, Stanford U.), XhoI digestion was performed, followed by a Klenow fill-in reaction, and a BamHI digest. These fragments were ligated to create pBMN-GFP-IP, which permits the expression of GFP and the puromycin resistance gene from a single bicistronic mRNA. pBMN-kapB-IP was generated by BamHI/EcoRI digestion of pCR3.1-kapB, releasing the 636 bp KapB ORF (derived from a pulmonary KS isolate). This fragment was subsequently ligated into the BamHI/EcoRI digested pBMN-IP vector. To create pBMN-MK2EE-IP, pcDNA3-Flag-MK2EE was amplified with primers G42/G43 from pcDNA3mycMK2T205ET317E [13]. The amplicons were digested with BamHI and XhoI, and inserted into BamHI/XhoI digested pBMN-IP vector. To create pBMN-hsp27DDD-IP, HA-hsp27DDD was excised from pcDNA3.1-HAhsp27DDD by sequential XbaI digest, Klenow fill-in to create a blunt end, and EcoRI digest. Following this, the insert was ligated into pBMN-IP, which had been prepared by sequential XhoI digest, Klenow fill-in to create a blunt end, and EcoRI digest. To generate pBMN-IP or pBMN-IB vectors containing RhoA-CA-eGFP and RhoA-DN-eGFP, the ORFs from pCB6-eGFP-RhoA-CA and pCB6-eGFP-RhoA-DN were amplified using Phusion High Fidelity Polymerase (NEB) chain reaction and the following primers: eGFP-N forward (5′-in database-3′) and reverse 5′-ATGCGAATTCTTATTATTACAAGACAAGGCACCCAGATT-3′. The resulting products were digested with BamHI and EcoRI and ligated into the pBMN-IP or pBMN-IB vector backbone. To generate HA-tagged versions of these clones, the ORFs of pCB6-eGFP-RhoA-CA and pCB6-eGFP-RhoA-DN were amplified using Phusion High Fidelity Polymerase (NEB) chain reaction and the following primers: forward 5′- GCATGGATCCACCATGGAGTACCCATACGATGTTCCAGATTACGCTCCCAGAGCTGCCATCCGGAAGAAAC-3′ and reverse 5′-ATGCGAATTCTTATTATTACAAGACAAGGCACCCAGATT-3′. The resulting PCR products were digested with BamHI and EcoRI and ligated into the pBMN-IP or pBMN-IB vector backbone. To create pBMN-IB-HA-Hsp27-AAA, the ORF of pCDNA3.1-HA-HSP27-AAA was amplified using Phusion High Fidelity Polymerase (NEB) chain reaction and the following primers: forward 5′- GGTGGAATTCATGGCTTACC-3′ and reverse 5′-ATGCCTCGAGTTATTATTACTTGGCGGCAGTCTCAT-3′. The resulting PCR product was digested with EcoRI and XhoI and ligated into the pBMN-IB vector backbone.

### shRNA retroviral plasmids

Retroviral shRNA expression vectors were created via PCR amplification of template oligonucleotides, and cloning into XhoI/EcoRI restriction sites in pSMP (Open Biosystems). Briefly, the following 97-mer template oligonucleotides were synthesized, sh-scrambled: TGCTGTTGACAGTGAGCGAGCACAAGCTGGAGTACAACTATAGTGAAGCCACAGATGTATAGTTGTACTCCAGCTTGTGCCTGCCTACTGCCTCGGA, shMK2: TGCTGTTGACAGTGAGCGCGCCTGAGAATCTCTTATACACTAGTGAAGCCACAGATGTAGTGTATAAGAGATTCTCAGGCTTGCCTACTGCCTCGGA. These sequences were PCR amplified with Xho pSMP forward primer (5′-CAGAAGGCTCGAGAAGGTATATTGCTGTTGACAGTGAGCG-3′) and Eco pSMP reverse primer (5′-CTAAAGTAGCCCCTTGAATTCCGAGGCAGTAGGCA-3′) and Pfu Ultra High Fidelity DNA polymerase (Stratagene). 110-bp amplicons were then digested with XhoI/EcoRI and ligated into XhoI/EcoRI digested pSMP retroviral vector.

### Lentiviral plasmids

pMD.2G (envelope) and pSPAX2 (packaging) plasmids were purchased from Addgene. All pGIPZ shRNA constructs used to knock down expression of the RhoA-specific guanine exchange factors (GEFs) listed below were purchased from Open Biosystems (oligo ID in parentheses): p115-3 (V3LHS_317458), p115-4 (V3LHS_317456), p115-9 (V2LHS_37090), H1-1 (V3LHS_317146), H1-2 (V3LHS_317143), and H1-7 (V2LHS_36680). pIPZ was created by PCR amplification of the enhanced CMV promoter of pGIPZ using a forward XbaI primer and a reverse NotI primer. After digestion of both the PCR product and pGIPZ vector with XbaI and NotI, the product was ligated into the vector backbone to create pIPZ. This new vector lacks the ORF for turbo eGFP and places the CMV promoter proximal to the IRES. To create pIPZ shRNA constructs used to knock down expression of the kaposin gene, we used previously generated shRNAs against kaposin in the pSM2 vector. Two different shRNA sequences that target the kaposin ORF were used and are named according to the position of the starting nucleotide. The 22-mer sequences for KapB 692 and KapB 746 shRNAs are 5′-TGTCCCGGATGTGTTACTAAAT-3′ and 5′-ACTCGTTTGTCTGTTGGCGATT-3′, respectively. In order to transfer these from pSM2 to pIPZ, the pSM2 vectors were digested with MluI and XhoI and inserted into pIPZ.

### Retrovirus and lentivirus preparation and infections

Retrovirus stocks were produced in 15-cm cell culture dishes by PEI-mediated transfection using 54 µl of PEI and 18 µg of the gene/shRNA of interest, contained in either the pBMN-IP, pBMN-IB or pSMP vector backbone, into the Phoenix amphotropic packing cell line (a kind gift from G. Nolan, Stanford). The transfection medium was replaced after 6 hours. Virus-containing supernatants were harvested 48 h after transfection. These supernatants were spinoculated onto target HUVEC cell monolayers for 2 h at 2,000 rpm in the presence of 5 µg/ml Polybrene (Sigma), and after 24 h, 1 µg/ml puromycin or 10 µg/ml blasticidin was added to select for transductants. Lentivirus stocks were produced in 15-cm cell culture dishes by PEI-mediated co-transfection of three plasmids: 10 µg of the shRNA of interest (in pGIPZ or pIPZ), 3 µg pMD.2G (envelope), 6 µg pSPAX2 (packaging) and 54 µl of PEI into HEK293T cells. Virus-containing supernatants were collected after 48 hours, diluted 1∶2, and added to target HUVEC monolayers for 4-5 hours at 37°C in the presence of 5 µg/ml polybrene (Sigma). After 24 h, 1 µg/ml puromycin was added to select for transductants.

### KSHV preparation and infections

Wild-type KSHV virus was produced from lytic reactivation of the BCBL-1 PEL cell line. Briefly, KSHV was induced to lytically reactivate from BCBL-1 cells at a cell concentration of 2×10^5^/ml using 0.3 mM valproic acid. After 7 days of induction, the suspension culture was pre-cleared by centrifugation for 10 minutes at 800×g before being filtered using 0.45 µm Millipore filters. The virus-containing filtrate was then centrifuged for 2 hours at 25,000×g and the supernatant discarded. The virus pellet was resuspended in 1/100 of the original culture volume of DMEM containing 10% FBS and stored at −80°C. Infectious viral titer was determined by immunofluorescent staining with anti-LANA (see method below). For the experiments reported in this paper, a 1∶25 dilution of stock virus was spinoculated onto HUVECs for 45 minutes at 2000 x g and 30°C without the addition of polybrene. The viral inoculum was left on the cells for an additional hour at 37°C before being replaced with normal EGM-2 media according to the methods of [87] [88]. Latently infected cells were fixed at the indicated times post infection.

### Luciferase assay

The luciferase reporter assay for identification of modulators of ARE-mediated mRNA decay is described in detail in [72]. Briefly, 10^5^ HeLa Tet-Off cells were cotransfected with 100 ng of a reporter plasmid master mix (pTRE2-Fluc-ARE and pTRE-2-Rluc, at a ratio of 9∶1); 900 ng of an expression vector or an empty vector control; and 3 µl of Fugene HD (Roche) according to the instructions of the manufacturer. Twenty-four hours after transfection, Dox was added (1 µg/ml) to stop *de novo* transcription from the pTRE reporter plasmids. Twenty-four hours after the addition of Dox, transfected cells were lysed in 200 µl of 1× passive lysis buffer, and samples were processed using the dual-luciferase assay kit (Promega) according to the instructions of the manufacturer. Firefly and Renilla luminescence was determined using the GloMax 20/20 luminometer (Promega). Firefly luminescent signal, expressed in relative light units (RLUs), was normalized to that of Renilla luciferase, to eliminate off-target effects of our expression plasmids or the transfection procedure. Results are expressed as normalized luciferase activity.

### Protein preparation and western blotting

Six-well plates of cells were washed once with phosphate-buffered saline and lysed directly in 1× sodium dodecyl sulfate (SDS) sample buffer. Equivalent amounts of protein (10 or 25 µg) were subjected to SDS-polyacrylamide gel electrophoresis and transferred to nitrocellulose membranes (Amersham). Membranes were blocked in Tris-buffered saline–Tween-20 (TBST) containing 5% bovine serum albumin unless otherwise indicated and probed overnight at 4°C using anti-KapB (a generous gift from D. Ganem used at 1∶5000 in 5% milk), anti-phospho(ser82)Hsp27 (1∶1000), anti-p115 (1∶1000), anti-H1 (1∶1000), anti-GAPDH (1∶1000, Abcam), anti-RhoA (1∶667), anti-MK2 (1∶1000) or anti-β-actin (1∶2500) antibody. Horseradish peroxidase-conjugated goat anti-rabbit and anti-mouse immunoglobulin secondary antibodies were used at a 1∶2000 dilution. All antibodies were purchased from Cell Signaling Technologies unless otherwise indicated. Secondary antibody was detected using ECL Plus detection reagents (Amersham Biosciences) according to the manufacturer's instructions. The chemiluminescent signal was detected using the Kodak Image Station 4000 mm PRO with no excitation or emission filter.

### Rho activation assay

6-well cluster dishes of HeLa-Tet Off cells were subjected to PEI-mediated transfection with 100 ng of a green fluorescent reporter protein, 1.0 µg of expression plasmids for KapB (pcr3.1 KapB), constitutively active MK2 (pcDNA3 FlagMK2-EE), phosphomimicking heat shock protein 27 (pcDNA3.1 HA-hsp27-DDD) or empty vector pcDNA3.1 and 3.3 µl of PEI per well. The transfection medium was replaced after 6 hours. 48 hours post transfection, 10^5^ transfected cells were seeded at sub-confluent density in a 10cm tissue culture dish. 24 hours later, cells were treated with starvation medium containing 0.1% FBS for 24 hours. The next day, cells were starved in medium without FBS for an additional 3–4 hours before being treated or not treated with LPA for 3 minutes. Alternately, confluent 6-well plates of either KapB- or vector control-expressing HUVECs were starved in low-serum EBM-2 medium for 24 hours and in serum-free medium for 4 hours before use. Cells were then immediately washed in ice-cold PBS, and lysed in 500 ul of 1 x Lysis buffer containing aprotinin, pepstatin, leupeptin, and PMSF on ice following the instructions of the Active Rho Detection Kit (Cell Signaling Technologies). Fresh lysates were clarified by centrifugation at 21,000 x g for 5 minutes at 4°C, kept cold at all times, and used immediately for active rho pull downs as recommended by the manufacturer. After binding for one hour, the unbound protein lysate was reserved and used for assay of total protein. For immunoblot analysis 10–20 µg of each total protein lysate and proportional amounts of Rho pull-down reaction (1∶10 ratio of lysate:pull-down) were subjected to 12%- SDS-polyacrylamide gel electrophoresis and transferred to nitrocellulose membranes (Amersham). Membranes were blocked Tris-buffered saline–Tween-20 (TBST) containing 5% bovine serum albumin and probed overnight at 4°C using anti-RhoA primary antibody (1∶667, CST) and horseradish peroxidase-conjugated goat anti-rabbit secondary antibody (1∶2000) and developed and imaged according to the above protocol.

### Immunofluorescent staining and microscopy

After transduction and selection, HUVECs were seeded on coverslips for microscopy. Twenty-four hours later, cells were either not treated or treated with the appropriate inhibitors or activators as described above and in the figure legends. After treatment, cells were fixed at room temperature in 4% paraformaldehyde (in PBS) for 10 min and then permeabilized with 0.1% Triton X-100 for 10 min. For staining microtubules, fixation was performed at 37°C in pre-warmed 4% paraformaldehyde (in D-PBS) at 37°C before permeabilization as indicated above. Cells were subsequently washed 3 times with PBS and then blocked in 1% human AB serum in PBS for 1 h at room temperature. To stain PB resident proteins, fixed cells were incubated with mouse anti-Hedls antibody (1∶1000; Santa Cruz) or rabbit anti-DDX6 antibody (C terminus, 1∶1000; Bethyl Laboratories) in 1% human AB serum overnight at 4°C. When appropriate and according to figure legends, cells were also incubated overnight at 4°C with rabbit anti-HA (1∶1600, CST), mouse anti-FLAG (1∶1600, Sigma), rabbit anti-LANA (1∶1000, a generous gift from D. Ganem), mouse anti-tubulin (1∶200, Santa Cruz) or rabbit anti-KapB antibody (1∶1000, a generous gift from D. Ganem) for 30 min at room temperature. Primary antibodies were removed by three 5-minute washes of PBS. Goat anti-rabbit Alexa 555, chicken anti-mouse Alexa 488, chicken anti-mouse Alexa 647 or goat anti-rabbit Alexa 647 secondary antibodies (Molecular Probes) were added for 1 h at room temperature in the dark. After being washed as described above, indicated cells were incubated with for one hour at room temperature with 1∶100 phalloidin in PBS (conjugated to either Alexa 555 or 647; Molecular Probes). Finally, cells were washed 3 more times and mounted on microscope slides with ProLong Gold antifade mounting medium (Invitrogen) and visualized using a Zeiss LSM 510 META laser-scanning confocal microscope and the 40× or 63x objective.

### Quantification of PBs

HUVECs transduced with vector were examined at 400x or 630x magnification, and the number of cells per field of view that contained normal-sized (approximately 300 nm in diameter; see [67]) PBs were counted. After counting between 100 and 200 cells (usually 4 fields of view), the percentage of cells containing normal PBs was determined. The effect of viral infection or ectopic expression on PB accretion was determined by counting only those cells that were positive by immune fluorescence for infection/gene expression. Results are displayed as the average fold reduction in cells with PBs compared to that of the untreated vector control (±SE).

### Wound healing assay

HUVEC cell monolayers were grown on gelatin-coated coverslips that had been etched with a reference marker, transduced with either the KapB retrovirus or an empty vector control and selected with puromycin as described above. After incubating cells in medium devoid of serum or growth factors for 1 hour, cell monolayers were wounded with a p200 pipette tip (by scraping off cells near to the reference marker), washed, and incubated in either complete media or media supplemented with VEGF (10 ng/ml). The ability of cells to repair the wound was monitored over time. Images at the time of wounding (t = 0) or at six hours (t = 6 hours) were captured using an Olympus CKX41 Inverted microscope, and the surface area (SA) of the initial and the remaining wound was determined using Image J. The percent of wound closure after the six hours was calculated using the equation SA(t = 0) –SA(t = 6)/SA(t = 0) ×100. Each experiment was performed in duplicate and the results presented are one representative experiment of three.

### Cell migration assays

Cell migration was assayed using a modified Boyden chamber assay [61]. HUVECs, transduced to express either KapB or an empty vector control, were harvested with trypsin, counted, centrifuged and resuspended in supplement-free EBM-2 medium containing 0.1% FBS (0.1%-EBM-2). 7.5×10^4^ cells were added to each 8.0 um pore size gelatinized polycarbonate membrane (Corning) separating the two chambers of a 6.5 mm transwell. After one hour of adhesion, either 0.1%-EBM-2 alone or media containing VEGF (1 or 10 ng/ml) was added to the lower chamber. After 4 hours, non-migratory cells remaining on the upper side of the membrane were removed by cotton swabbing and the cells on the underside of the membrane were fixed with 4% paraformaldehyde before staining with 0.2% crystal violet for 1–2 hours. The filters were dried, removed from the chambers, and visualized by microscopic examination. The number of migrated cells on the lower face of the filter was counted in five random fields at 400x magnification. Assays were done in duplicate and were repeated in three independent experiments. The data represents the average of two technical duplicates and three independent experiments +/− SE.

### Tubule formation assays

Wells of a 48-well plate were coated with 200 µl Matrigel (BD Biosciences). HUVECs, transduced to express KapB, or constitutively active MK2 (MK2-EE) or an empty vector control, were harvested with trypsin, counted, centrifuged and resuspended in basal EBM-2 medium. 5×10^4^ cells were added to the top of each matrigel-containing well in basal media. Cells were incubated at 37°C and observed every hour. Over time, HUVECs progress from individual cells to connected tubules by first forming sprouted cells and then connections between small groups of cells. As tubulogenesis progresses, cells form connected tubes, enclosed polygons and complex meshwork (layered tubes of cells) as described in [66]. At 5 hours, extensive tubules, often with the presence of polygons and complex mesh, had formed and were visualized using an Olympus CKX41 inverted microscope. Representative images were captured using Image Pro Plus. To quantify the angiogenic potential of KapB, an angiogenic score was calculated by adapting the methods of [66]. For each condition, 5 random fields of view at 200x magnification were observed and the number of enclosed polygons was counted. Further, the presence of a complex meshwork was given the following score: 1 =  no complex mesh, 2 =  presence of complex mesh, and 3 =  complex mesh of cell thickness >4 cells. The angiogenic score was then calculated as the product of the number of enclosed polygons and the complex mesh score. Assays were done in duplicate and were repeated in three independent experiments. The data represents the average of two technical duplicates and three independent experiments +/− SE.

### Statistical analysis

Graphing and statistical analyses were performed using GraphPad Prism software. All data are presented as mean +/− SEM of three independent experiments unless otherwise indicated. Paired parametric t-test was used for comparison between two groups. One-way repeated measures ANOVA was used for comparison between multiple groups where the mean of each group was compared to the mean of the control group. P values are displayed when p<0.05.

## Supporting Information

S1 Fig **KapB-mediated actin stress fiber formation is a cell autonomous effect.** HUVECs expressing either KapB (panels b, d, f, h) or an empty vector control (panels a, c, e, g) were starved for two hours in media lacking serum and growth factors and then treated for two hours with either complete serum media (c–d), conditioned media taken from previously transduced HUVECs expressing either KapB (g–h) or a vector control (e–f), or starved for an additional two hours (a–b). These cells were then fixed and stained with Alexa 555-conjugated phalloidin to visualize actin stress fibers. Scale bars  = 10 µm.
(TIF)

S2 Fig **KapB expression enhances endothelial cell migration.** A) HUVECs expressing KapB (b, d) or an empty vector control (a, c) were grown to near confluence on etched coverslips before wounding the cell monolayer using a p200 tip. The ability of the cells to repair the wound over time was monitored and quantified using Image J. Images of the wounded monolayers were captured at the time of wounding (panels a–b) and after 6 hours (panels c–d). One representative experiment of three is shown. B) Cell migration was assayed using a modified Boyden chamber assay [61]. HUVECs, transduced to express either KapB or an empty vector control, were harvested with trypsin, counted, centrifuged and resuspended in supplement-free EBM-2 medium containing 0.1% FBS (0.1%-EBM-2). 7.5×10^4^ were added to each 8.0 µm pore size gelatinized polycarbonate membrane separating the two chambers of a 6.5 mm transwell. After one hour of adhesion, either 0.1%-EBM-2 alone or media containing VEGF (1 or 10 ng/ml) was added to the lower chamber. After 4 hours, non-migratory cells remaining on the upper side of the membrane were removed by cotton swabbing and the cells on the underside of the membrane were fixed with 4% paraformaldehyde before staining with 0.2% crystal violet. The number of migrated cells on the lower face of the filter was counted in five random fields at 400x magnification. Assays were done in duplicate and data represents the average + standard error from three independent experiments.
(TIF)

S3 Fig **Kaposin B expression is detected throughout various treatments and during latent KSHV infection of HUVECs.** A) KSHV clones have been derived from several different isolates of KS and these viruses express multiple different isoforms of KapB. Our recombinant retrovirus expression plasmids express the 25 kDa form of KapB originally isolated from KSHV-infected pulmonary KS. Our wild-type KSHV stocks are derived from the primary effusion lymphoma (PEL) BCBL-1 cell line, and express the 48 kDa isoform of KapB. Due to the complex translational program of the kaposin locus, multiple other Kaposin translation products are also typically observed. B-C) HUVECs were transduced with recombinant retroviruses that express KapB or vector (V) control (B) or infected with KSHV (two independently produced stocks) for 72 hours (C). Following two-day selection with puromycin, transduced cells were either treated with lysophosphatidic acid (LPA), vascular endothelial growth factor (VEGF) or not treated for 3 minutes (LPA) or one hour (VEGF). After treatment, cells were lysed in 1x SDS-protein sample buffer containing protease inhibitors and processed for SDS-PAGE and immunoblotting using anti-KapB and anti-beta-actin. One representative experiment of two is shown.
(TIF)

S4 Fig **KapB expression enhances angiogenesis in a tubule formation assay.** Wells of a 48-well plate were coated with Matrigel. HUVECs, transduced to express KapB, MK2-EE or an empty vector control, were harvested with trypsin, counted, centrifuged and resuspended in basal EBM-2 medium. 5×10^4^ cells were added to the top of each matrigel-containing well in serum-free basal media with or without the addition of a chemical inhibitor of rho kinase ROCK1/2 (10 µM of Y-27632). The ability of these cells to sprout, form connections, and following that form connected tubules, enclosed polygons and complex meshwork was monitored over time. A) At 5 hours, extensive tubules, often with the presence of polygons and complex mesh, formed and representative phase contrast microscope images were captured. B) An angiogenic score was calculated as follows. For each condition, 5 random fields of view at 200x magnification were visualized the angiogenic potential was calculated (angiogenic score  =  # polygons x complex meshwork score 1, 2 or 3). The angiogenic potential of each condition was quantified from duplicate wells per experiment and is expressed as the average of five independent experiments +/− the standard error.
(TIF)

S5 Fig **Knockdown of p115RhoGEF and GEF H1 in HUVECs.** A–B) HUVECs were transduced with recombinant GFP-expressing lentiviruses that express short hairpin RNAs (shRNAs) against the Rho guanine exchange factors (GEFs; p115 [numbered −3, −4, and −9] and H1 [numbered −1, −2]) or the non-specific (NS) shRNA control. Positive transductants were selected by puromycin treatment and positive GFP-expression. After re-seeding cells in 6-well plates for 24 hours, transduced cells were washed with PBS and lysed in 1x SDS-protein sample buffer containing protease inhibitors and processed for SDS-PAGE and immunoblotting using anti-p115RhoGEF, anti-GEF H1 and anti-GAPDH. One representative blot of three independent experiments is shown.
(TIF)

S6 Fig **Knockdown of the Rho guanine exchange factor (GEF) p115 reduces MK2-EE and hsp27-DDD-induced modification of p-body dynamics.** A-B) HUVECs were sequentially transduced with two populations of recombinant viruses: firstly, puromycin-resistant viruses that express MK2 EE-Flag, hsp27 DDD-HA or the empty vector; and secondly, GFP-expressing lentiviruses that express short hairpin RNAs (shRNAs) against a two different Rho guanine exchange factors (GEFs; p115 [numbered −3, −4, and −9], H1 [numbered −1, −2, and −7], or the non-specific (NS) shRNA control. Positive transductants were selected by puromycin treatment (1^st^ step) and positive GFP-expression, to mark shRNA-expressing cells (2^nd^ step). After seeding cells on coverslips, and a one-hour treatment in basal media the following day, cells were stained with the following primary antibodies: mouse anti-hedls or rabbit anti-DDX6 (to stain p-bodies, false-colored blue), rabbit anti-HA (to stain hsp27-DDD-HA, red), or mouse anti-Flag (to stain MK2-EE-Flag, red). Representative IF images are shown in A. To quantify p-body disruption, the number of cells expressing the transgene of interest (red) and the shRNA construct (green) that retained normal p-bodies was counted as for Fig. 4 and shown in B (n = 3 independent experiments). Scale bar  = 10 µm.
(TIF)
