# Supplementary figures and images for: Viral Activation of MK2-hsp27-p115RhoGEF-RhoA Signaling Axis Causes Cytoskeletal Rearrangements, P-body Disruption and ARE-mRNA Stabilization
Source: PLoS Pathog. 2015 Jan 8;11(1):e1004597. doi: 10.1371/journal.ppat.1004597 (PMC4287613; doi:10.1371/journal.ppat.1004597)

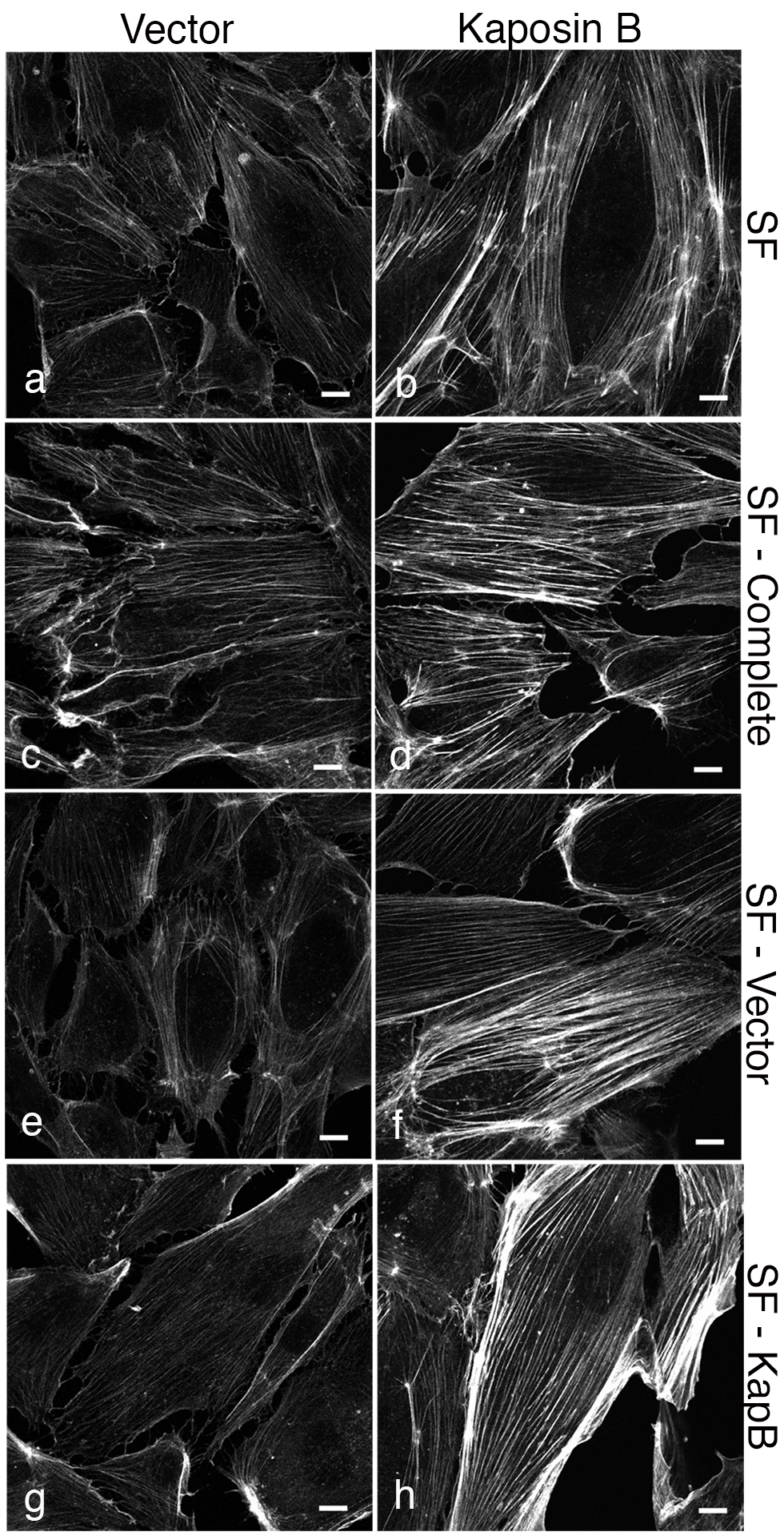

Supplement: S1 Fig — KapB-mediated actin stress fiber formation is a cell autonomous effect. HUVECs expressing either KapB (panels b, d, f, h) or an empty vector control (panels a, c, e, g) were starved for two hours in media lacking serum and growth factors and then treated for two hours with either complete serum media (c–d), conditioned media taken from previously transduced HUVECs expressing either KapB (g–h) or a vector control (e–f), or starved for an additional two hours (a–b). These cells were then fixed and stained with Alexa 555-conjugated phalloidin to visualize actin stress fibers. Scale bars = 10 µm. (TIF) [file ppat.1004597.s001.tif]

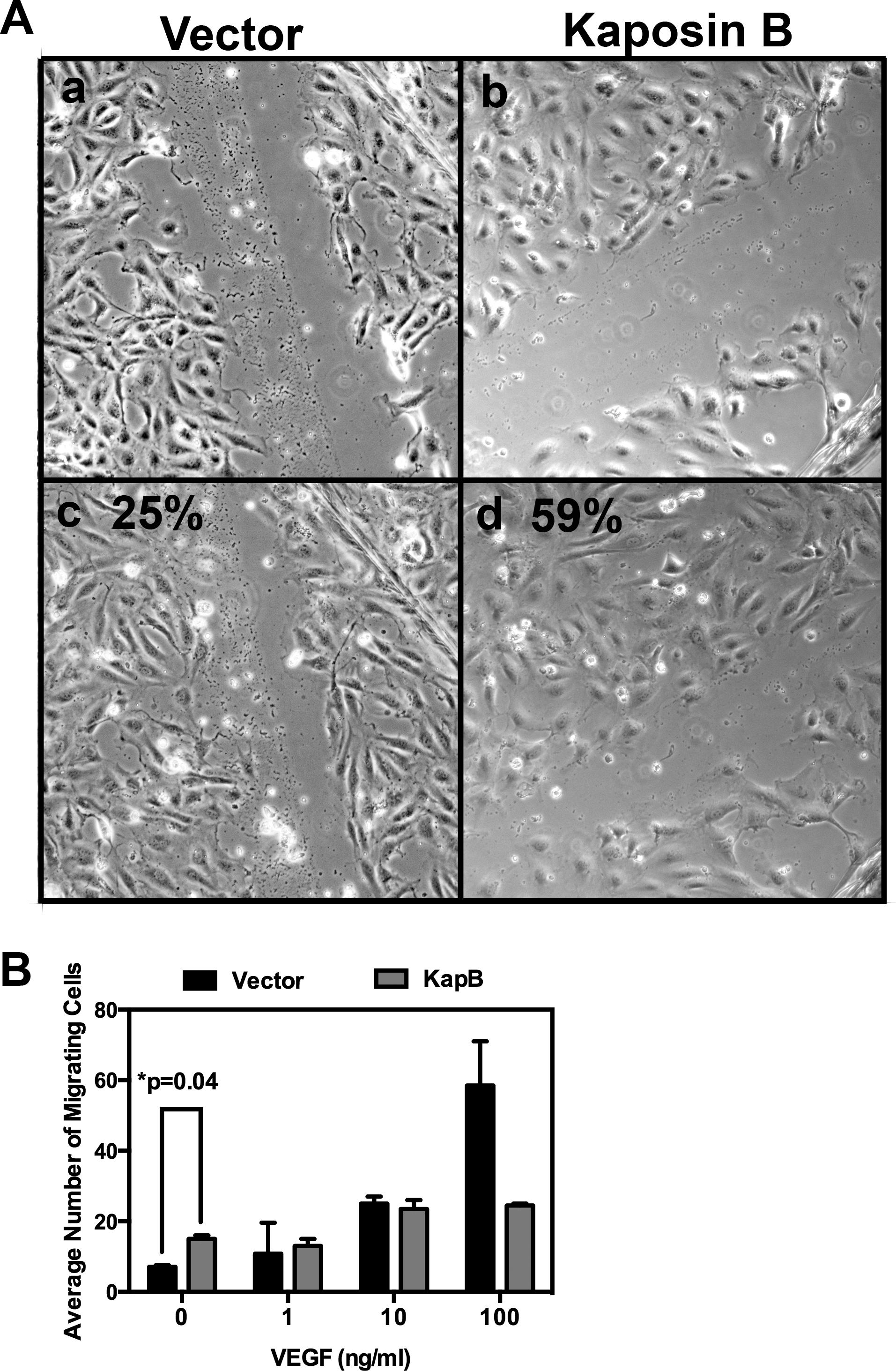

Supplement: S2 Fig — KapB expression enhances endothelial cell migration. A) HUVECs expressing KapB (b, d) or an empty vector control (a, c) were grown to near confluence on etched coverslips before wounding the cell monolayer using a p200 tip. The ability of the cells to repair the wound over time was monitored and quantified using Image J. Images of the wounded monolayers were captured at the time of wounding (panels a–b) and after 6 hours (panels c–d). One representative experiment of three is shown. B) Cell migration was assayed using a modified Boyden chamber assay [61]. HUVECs, transduced to express either KapB or an empty vector control, were harvested with trypsin, counted, centrifuged and resuspended in supplement-free EBM-2 medium containing 0.1% FBS (0.1%-EBM-2). 7.5×10^4^ were added to each 8.0 µm pore size gelatinized polycarbonate membrane separating the two chambers of a 6.5 mm transwell. After one hour of adhesion, either 0.1%-EBM-2 alone or media containing VEGF (1 or 10 ng/ml) was added to the lower chamber. After 4 hours, non-migratory cells remaining on the upper side of the membrane were removed by cotton swabbing and the cells on the underside of the membrane were fixed with 4% paraformaldehyde before staining with 0.2% crystal violet. The number of migrated cells on the lower face of the filter was counted in five random fields at 400x magnification. Assays were done in duplicate and data represents the average + standard error from three independent experiments. (TIF) [file ppat.1004597.s002.tif]

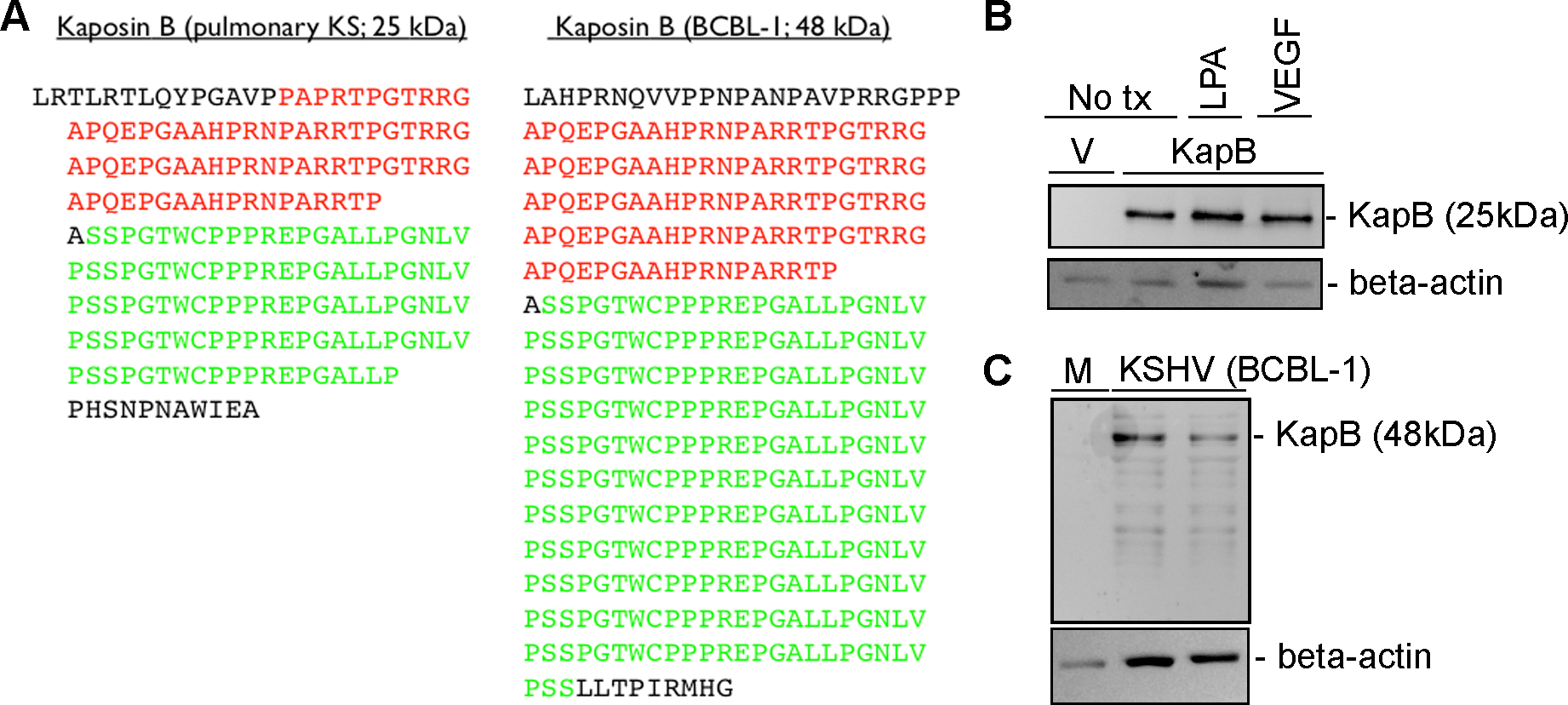

Supplement: S3 Fig — Kaposin B expression is detected throughout various treatments and during latent KSHV infection of HUVECs. A) KSHV clones have been derived from several different isolates of KS and these viruses express multiple different isoforms of KapB. Our recombinant retrovirus expression plasmids express the 25 kDa form of KapB originally isolated from KSHV-infected pulmonary KS. Our wild-type KSHV stocks are derived from the primary effusion lymphoma (PEL) BCBL-1 cell line, and express the 48 kDa isoform of KapB. Due to the complex translational program of the kaposin locus, multiple other Kaposin translation products are also typically observed. B-C) HUVECs were transduced with recombinant retroviruses that express KapB or vector (V) control (B) or infected with KSHV (two independently produced stocks) for 72 hours (C). Following two-day selection with puromycin, transduced cells were either treated with lysophosphatidic acid (LPA), vascular endothelial growth factor (VEGF) or not treated for 3 minutes (LPA) or one hour (VEGF). After treatment, cells were lysed in 1x SDS-protein sample buffer containing protease inhibitors and processed for SDS-PAGE and immunoblotting using anti-KapB and anti-beta-actin. One representative experiment of two is shown. (TIF) [file ppat.1004597.s003.tif]

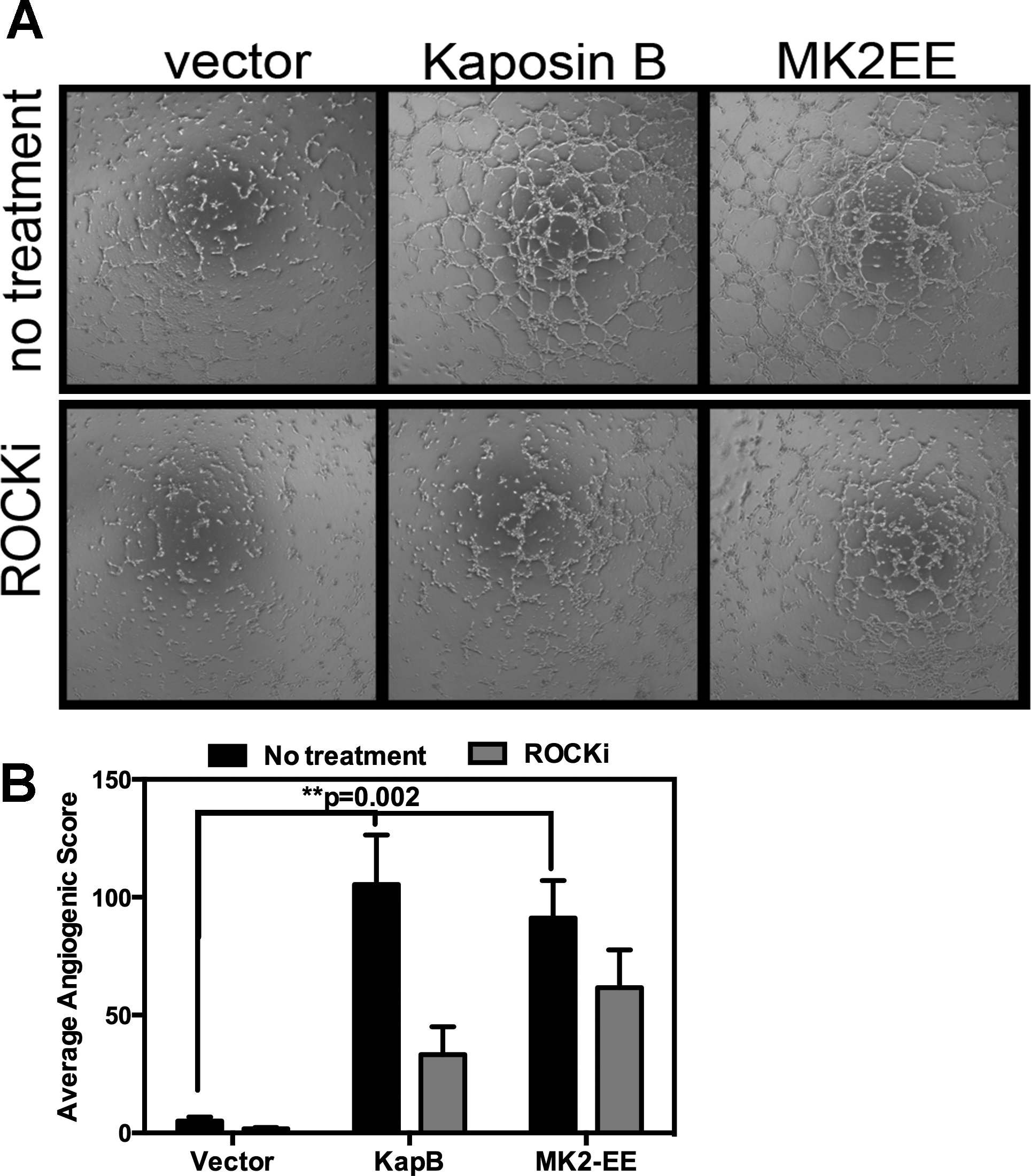

Supplement: S4 Fig — KapB expression enhances angiogenesis in a tubule formation assay. Wells of a 48-well plate were coated with Matrigel. HUVECs, transduced to express KapB, MK2-EE or an empty vector control, were harvested with trypsin, counted, centrifuged and resuspended in basal EBM-2 medium. 5×10^4^ cells were added to the top of each matrigel-containing well in serum-free basal media with or without the addition of a chemical inhibitor of rho kinase ROCK1/2 (10 µM of Y-27632). The ability of these cells to sprout, form connections, and following that form connected tubules, enclosed polygons and complex meshwork was monitored over time. A) At 5 hours, extensive tubules, often with the presence of polygons and complex mesh, formed and representative phase contrast microscope images were captured. B) An angiogenic score was calculated as follows. For each condition, 5 random fields of view at 200x magnification were visualized the angiogenic potential was calculated (angiogenic score = # polygons x complex meshwork score 1, 2 or 3). The angiogenic potential of each condition was quantified from duplicate wells per experiment and is expressed as the average of five independent experiments +/− the standard error. (TIF) [file ppat.1004597.s004.tif]

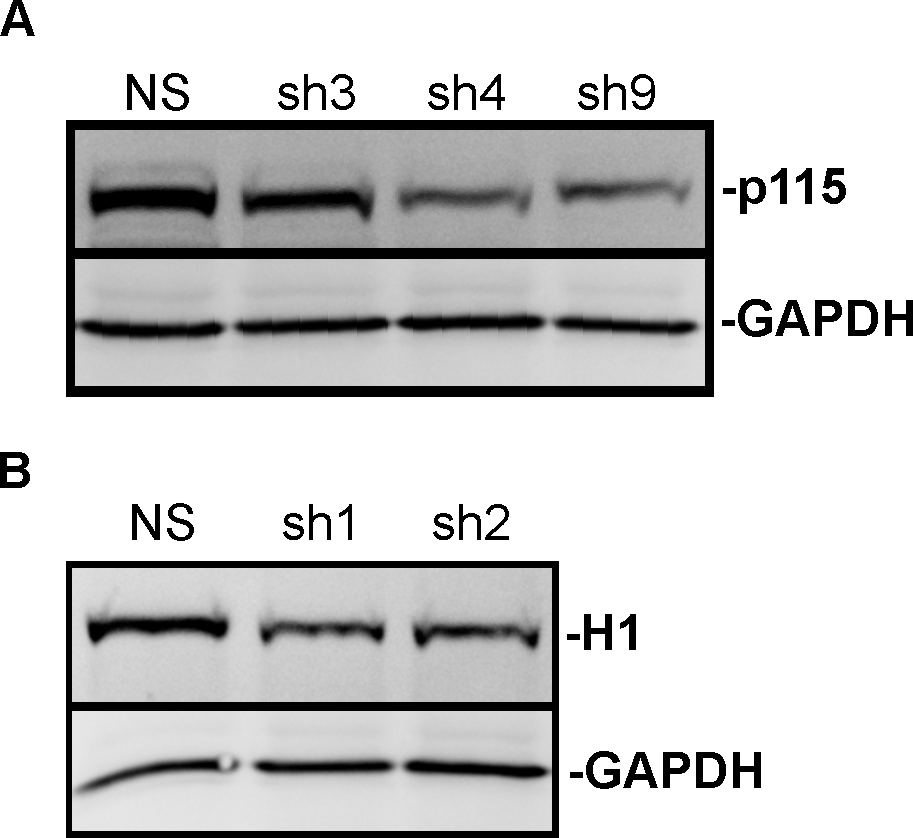

Supplement: S5 Fig — Knockdown of p115RhoGEF and GEF H1 in HUVECs. A–B) HUVECs were transduced with recombinant GFP-expressing lentiviruses that express short hairpin RNAs (shRNAs) against the Rho guanine exchange factors (GEFs; p115 [numbered −3, −4, and −9] and H1 [numbered −1, −2]) or the non-specific (NS) shRNA control. Positive transductants were selected by puromycin treatment and positive GFP-expression. After re-seeding cells in 6-well plates for 24 hours, transduced cells were washed with PBS and lysed in 1x SDS-protein sample buffer containing protease inhibitors and processed for SDS-PAGE and immunoblotting using anti-p115RhoGEF, anti-GEF H1 and anti-GAPDH. One representative blot of three independent experiments is shown. (TIF) [file ppat.1004597.s005.tif]

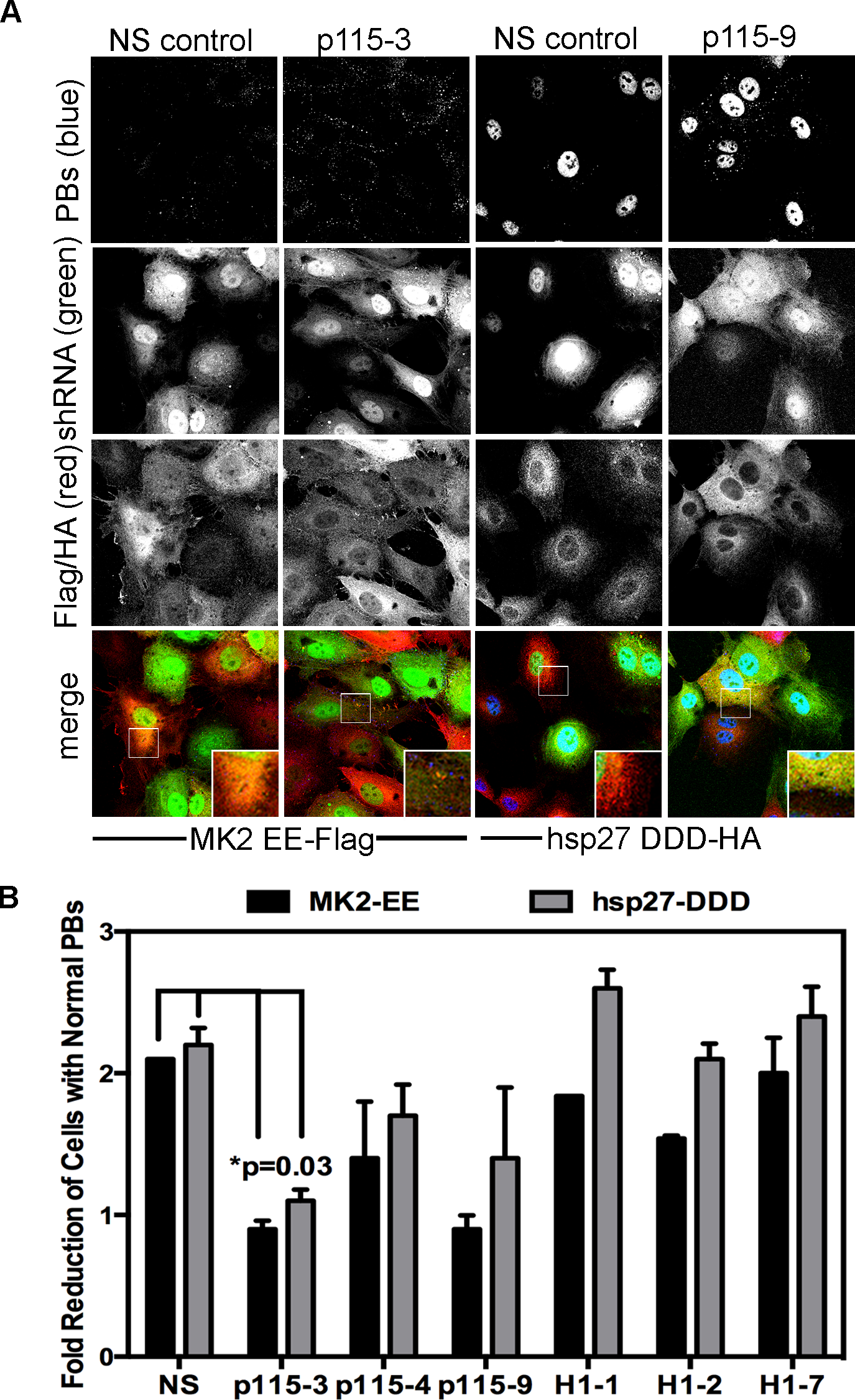

Supplement: S6 Fig — Knockdown of the Rho guanine exchange factor (GEF) p115 reduces MK2-EE and hsp27-DDD-induced modification of p-body dynamics. A-B) HUVECs were sequentially transduced with two populations of recombinant viruses: firstly, puromycin-resistant viruses that express MK2 EE-Flag, hsp27 DDD-HA or the empty vector; and secondly, GFP-expressing lentiviruses that express short hairpin RNAs (shRNAs) against a two different Rho guanine exchange factors (GEFs; p115 [numbered −3, −4, and −9], H1 [numbered −1, −2, and −7], or the non-specific (NS) shRNA control. Positive transductants were selected by puromycin treatment (1st step) and positive GFP-expression, to mark shRNA-expressing cells (2nd step). After seeding cells on coverslips, and a one-hour treatment in basal media the following day, cells were stained with the following primary antibodies: mouse anti-hedls or rabbit anti-DDX6 (to stain p-bodies, false-colored blue), rabbit anti-HA (to stain hsp27-DDD-HA, red), or mouse anti-Flag (to stain MK2-EE-Flag, red). Representative IF images are shown in A. To quantify p-body disruption, the number of cells expressing the transgene of interest (red) and the shRNA construct (green) that retained normal p-bodies was counted as for Fig. 4 and shown in B (n = 3 independent experiments). Scale bar = 10 µm. (TIF) [file ppat.1004597.s006.tif]
